# Single-molecule magnetostrictor: an {Fe_8_Gd_8_} cubic crystal exhibits temperature-dependent magnetostriction

**DOI:** 10.1093/nsr/nwag300

**Published:** 2026-05-21

**Authors:** Dong-Yang Li, Lei Qin, Yuan-Qi Zhai, Jia-Shu Sun, Xia-Li Ding, Jin-Tao Lu, Ismael Francisco Díaz-Ortega, Hiroyuki Nojiri, Zhendong Fu, Yan-Zhen Zheng

**Affiliations:** Frontier Institute of Science and Technology, Interdisciplinary Research Center of Frontier Science and Technology, State Key Laboratory of Electrical Insulation and Power Equipment, Xi’an Key Laboratory of Electronic Devices and Materials Chemistry, Xi’an Jiaotong University, Xi’an 710054, China; Frontier Institute of Science and Technology, Interdisciplinary Research Center of Frontier Science and Technology, State Key Laboratory of Electrical Insulation and Power Equipment, Xi’an Key Laboratory of Electronic Devices and Materials Chemistry, Xi’an Jiaotong University, Xi’an 710054, China; Frontier Institute of Science and Technology, Interdisciplinary Research Center of Frontier Science and Technology, State Key Laboratory of Electrical Insulation and Power Equipment, Xi’an Key Laboratory of Electronic Devices and Materials Chemistry, Xi’an Jiaotong University, Xi’an 710054, China; Frontier Institute of Science and Technology, Interdisciplinary Research Center of Frontier Science and Technology, State Key Laboratory of Electrical Insulation and Power Equipment, Xi’an Key Laboratory of Electronic Devices and Materials Chemistry, Xi’an Jiaotong University, Xi’an 710054, China; Shaanxi Science and Technology Holding Institute, Xi’an 710016, China; Key Laboratory of the Ministry of Education and International Center for Dielectric Research, School of Electronic Science and Engineering, Xi’an Jiaotong University, Xi’an 710049, China; Xi’an SIMOLDE Science and Technology Co., Ltd, Xi’an 710049, China; Institute for Materials Research (IMR), Tohoku University, Sendai 980-8577, Japan; Departamento de Química y Física-CIESOL, Universidad de Almería, Almería 04120, Spain; Institute for Materials Research (IMR), Tohoku University, Sendai 980-8577, Japan; Songshan Lake Materials Laboratory, Dongguan 523808, China; Frontier Institute of Science and Technology, Interdisciplinary Research Center of Frontier Science and Technology, State Key Laboratory of Electrical Insulation and Power Equipment, Xi’an Key Laboratory of Electronic Devices and Materials Chemistry, Xi’an Jiaotong University, Xi’an 710054, China

**Keywords:** magnetostriction, iron(III), gadolinium(III), molecule, exchange-coupling

## Abstract

Molecular magnets, as an important category of nanosized magnetic materials have attracted considerable interest for their wide applications such as contrasting agents for magnetic resonance imaging, low-temperature magnetic refrigerants, and spintronic devices. Up to date, though magnet-type behaviour has been enhanced to 100 K for single-molecule magnets, the investigation of molecule-originated magnetostriction (MS) is still in its infancy. Here, we report the observation of a large MS effect (50 ppm at 2 K under a field of 7 T) in a crystal solid of the wheel-like {Fe_8_Gd_8_} coordination molecules. The {Fe_8_Gd_8_} molecules crystallized in a highly symmetric cubic space group of $Pn\bar{3}n$ and have no long-range magnetic ordering down to 0.2 K. Therefore, we are convinced that such a giant magnetostrictive effect for {Fe_8_Gd_8_} is a single-molecule origin. We further used quantum Monte Carlo simulation to fit the temperature-dependent magnetostriction data and gave an excellent overlap with *J*_Fe–Gd_ = 4.81(5) K. Such a strong ferromagnetic interaction between Fe^3+^ and Gd^3+^ ensures a large magnetic momentum for {Fe_8_Gd_8_}, which is the key source of magnetoelastic response to the external field at low temperatures. Therefore, this work explicitly demonstrates that predominantly intramolecular exchange-coupling can cause magnetostriction, inspiring the design of new magnetostrictive materials.

## Teaser

This work reports an unambiguous molecular origin of large magnetostriction (50 ppm at 2 K and Δ*H* = 7 T) for a cubic crystal of the {Fe_8_Gd_8_} wheels driven by predominantly intramolecular ferromagnetic exchange-coupling interactions.

## INTRODUCTION

Molecular magnets have captivated the scientific community since the discovery of the archetypal {Mn_12_} complex [1]. Unlike traditional bulk magnets based on long-range ordering of metallic spins, molecular magnets are discrete molecules that exhibit magnetic bistability and slow relaxation of magnetization of molecular origin. Over the past three decades, these systems have served as ideal playgrounds for exploring quantum phenomena, such as quantum tunnelling of magnetization [2], while promising revolutionary applications in high-density data storage, quantum information processing (qubits), and molecular spintronics [3,4]. Recently, the working temperature scope of single-molecule magnets has been raised to 100 K, well above the boiling point of liquid nitrogen [5]. Moreover, notably application of molecule-based materials as cryogenic magnetic refrigerants has also been demonstrated [6]. Recently, the macroscopic mechanical response of molecule-based material under external magnetic field variation has also been reported, but the overall cases are still very limited and the mechanism behind this phenomenon remains elusive [7–9]. This gap has limited a complete understanding of magneto-elastic coupling at the molecular level and missed the opportunity to develop novel smart materials through molecular design.

Magnetostriction (MS), the phenomenon whereby magnetic materials undergo shape or dimensional changes during magnetization, has been utilized across various areas such as micro-displacement control, precision machinery, and modern robotics [10–12]. In traditional ferromagnetic or ferrimagnetic materials, the mechanism of MS is generally attributed to the collective translation and/or rotation of magnetic domains, which are reoriented by an external field (Fig. S1A) [13–16,40]. Large MS effects have been observed in some paramagnetic rare-earth (RE) materials due to the crystal field effect (Fig. S1B). For instance, significant anisotropic thermal expansion driven by crystal field splitting has been experimentally verified in intermetallic compounds such as ErCu_2_ and NdCu_2_ [17]. Recently, the paramagnetic KEr(MoO_4_)_2_ salt shows 400 ppm at 2.9 K under an applied magnetic field of 12 T [18]. Although these mechanisms are well-established in inorganic materials [19,20], direct evidence has been lacking in the field of molecular magnets, particularly for MS driven by intramolecular exchange coupling with macroscopically measurable effects. Early observations of field-dependent exchange interactions in molecular clusters such as {Ni_4_Mo_12_} hinted at field-induced structural distortions [21], but the direct observation of macroscopic temperature-dependent MS driven by unambiguous intramolecular exchange-coupling interaction remains unreported.

In this context, our previous designed 3d–4f mixed metal molecular wheels, named {*M*_8_RE_8_} (*M*^3+^ = Cr^3+^, Fe^3+^, Al^3+^, Sc^3+^; RE^3+^ = Gd^3+^, Tb^3+^, Dy^3+^, Er^3+^, Y^3+^), offer an ideal platform for investigation of molecule origin MS [22–26]. The alternative metal centres allow precise selection of different spins: for instance, the use of metal ions with quenched orbital angular momentum such as Fe^3+^ (*S* = 5/2 and *L* = 0) and Gd^3+^ (*S* = 7/2 and *L* = 0) can significantly suppress interference from single-ion anisotropy (crystal-field effect), while the introduction of diamagnetic ions such as Al^3+^, Sc^3+^, and Y^3+^ to prepare analogous compounds enables the isolation of pure exchange interaction contributions through comparative studies [27–33]. Moreover, such 3d–4f mixed metal molecular wheels tend to crystallize in highly symmetric space group, such as cubic $Pn\bar{3}n$, which further offers the opportunity to significantly suppress crystalline anisotropy. Therefore, the dominant effect of intramolecular exchange-coupling interactions on MS can be elucidated.

Herein, we report the observation of single-molecule origin of MS effect in a heterometallic molecular wheel, the [Fe_8_Gd_8_(mdea)_16_(CH_3_COO)_16_]·CH_3_CN·10H_2_O (abbr. {Fe_8_Gd_8_}), where mdeaH_2_ = *N*-methyldiethanolamine. We observed a significant MS effect below 50 K. Such an effect is growing more evident upon cooling, reaching a maximum of 50 ppm at 7 T and 2 K. We further use quantum Monte Carlo (QMC) simulation to retrieve the exchange-coupling constants from the magnetic and high-field/frequency electron paramagnetic resonance (HF-EPR) spectroscopy by comparing the diamagnetic counterparts. Finally, we used the obtained exchange-coupling constants to verify the theoretical model, leading to the agreement between the experimental data and the theoretical simulation. As there is also no long-range magnetic ordering observed from the heat capacity data down to 0.2 K, which excludes the possibility of domain wall movements. Thus, this finding unveils a single-molecule origin for MS materials and may inspire the design of new generation MS materials based on molecules.

## RESULTS AND DISCUSSION

### Crystal structure

**Figure 1.**
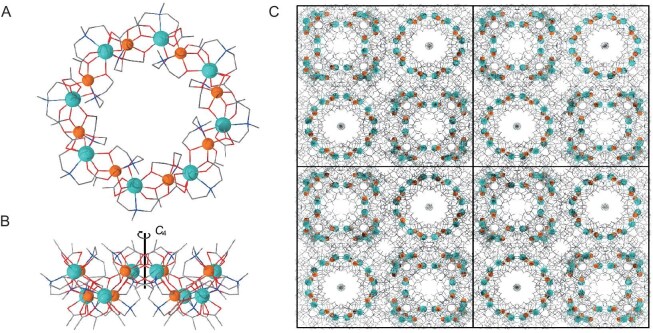
Molecular structure of {Fe_8_Gd_8_}. (A) Ball-and-stick model of the molecular structure of compound {Fe_8_Gd_8_} viewed along the *b* axis. (B) Ball-and-stick model of the molecular structure of compound {Fe_8_Gd_8_} viewed along the *a* axis. (C) Molecular packing of {Fe_8_Gd_8_} in 2 × 2 unit cells. Colour code: Fe, orange; Gd, sky blue; O, red; N, deep blue; and C, grey. The hydrogen atoms are omitted for clarity.

Single crystal X-ray diffraction revealed that {Fe_8_Gd_8_} molecules crystallize in the cubic space group $Pn\bar{3}n$ (No. 222), characterized by a primitive lattice, centrosymmetric, 3-fold rotoinversion axes and glide planes, in full agreement with the observed symmetry elements and lattice parameters. Figure S3 illustrates the symmetry elements and the unit cell packing arrangement defined by the space group, which is consistent with the highly symmetric nature of the molecular lattice. The asymmetric unit contains a molecular ring composed of eight Fe^3+^ and eight Gd^3+^ cations linked by organic ligands, forming a wheel-like structure with a crystallographic *C*_4_ axis (Fig. 1A and B). Each Fe^3+^ cation is six-coordinate, bonded to three mdea^2−^ ligands and one acetate anion. Each Gd^3+^ cation is eight-coordinate, sharing the same ligands as Fe^3+^ cation and binding one additional terminal acetate. Intermetallic Fe–Gd distances are 3.39–3.48 Å, while average Fe–O and Gd–O distances are 1.95–2.06 and 2.32–2.48 Å, respectively. The molecular wheels adopt a face-to-face packing arrangement, forming three types of double-ring cubic lattices, while they are periodically arranged to generate the cubic crystal (Fig. 1C). This cubic packing enforces isotropic environments, thereby providing a structural basis for minimizing crystal-field-driven magnetostriction.

### Magnetic properties and exchange coupling

The temperature-dependent magnetic susceptibility data of a polycrystalline {Fe_8_Gd_8_} sample was measured over the range 2–300 K. No evidence of long-range magnetic ordering was observed across the entire temperature range; thus, the magnetic properties reflect the characters of single molecules. At room temperature, the effective magnetic moment (*μ*_eff_) is 28.2 *μ*_B_, in close agreement with the expected value of 28 *μ*_B_ for eight spin-only Gd^3+^ and eight spin-only Fe^3+^ ions. Upon cooling, *μ*_eff_ remains nearly constant until 100 K, followed by a rapid increase to about 8 K with the maximum value of 31.2 *μ*_B_. Then, an abrupt decrease is seen till 2 K, where the *μ*_eff_ is 26.2 *μ*_B_ (Fig. S6). The Curie–Weiss fitting of the *χ*^−1^ vs. *T* plot from 100 to 300 K affords *C* = 101.6 cm^3^ mol^−1^ K and *θ* = 0.82 K, the positive *θ* indicates ferromagnetic interactions between Fe^3+^ and Gd^3+^ metal centres (Fig. S7).

**Figure 2.**
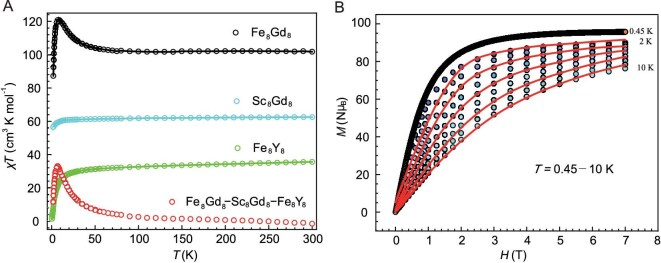
Magnetization measurements. (A) Temperature-dependent product of direct current (DC) magnetic susceptibility and temperature measured under a 0.1 T field from 2 to 300 K for {Sc_8_Gd_8_}, {Fe_8_Y_8_}, {Fe_8_Gd_8_}, and data for {Fe_8_Gd_8_} derived by subtracting the contributions of {Fe_8_Y_8_} and {Sc_8_Gd_8_}. (B) Field dependence of magnetization plots at 0.45–10 K. The curves are fitted using the Directed Loop algorithm within the ALPS package. For the 0.45 K data, the plot represents the average of the up and down sweep measurements, with the complete raw data provided in Fig. S13.

To elucidate the magnetic interactions in the {Fe_8_Gd_8_} system, particularly the Fe^3+^–Gd^3+^ coupling, this study employed a subtraction approach using isostructural diamagnetic ions substituted analogous compounds (that is, {Fe_8_Y_8_} and {Sc_8_Gd_8_}) as magnetic ‘background’, a strategy successfully employed in solving complex couplings in high-nuclearity 3d-4f clusters [22,23,34]. By systematically subtracting the magnetic susceptibility data of {Fe_8_Y_8_} and {Sc_8_Gd_8_} (the former contains Fe–Fe interaction and individual Fe^3+^ ions and the latter contains Gd−Gd interactions and individual Gd^3+^ ions) from that of {Fe_8_Gd_8_}, the net contribution from Fe^3+^–Gd^3+^ exchange-coupling can be obtained (Fig. 2A). Previously, the Fe^3+^–Fe^3+^ and Gd^3+^–Gd^3+^ exchange-coupling constants were determined, namely *J*_Fe–Fe_ = −0.13(4) K (−0.1 cm^−1^) and *J*_Gd–Gd_ = −0.013 K (−0.009 cm^−1^) [22,23]_._ Through a systematic comparative analysis against the isostructural analogues, the contributions from homometallic interactions were deconvoluted. Consequently, within our theoretical framework, the distinct phenomenological behaviour of the Fe^3+^–Gd^3+^ magnetic coupling can be unambiguously isolated and elucidated. This subtraction method revealed the upward curvature upon cooling, providing unequivocal evidence for ferromagnetic coupling between Fe^3+^ and Gd^3+^ ions. The magnetization plots (*M*) from 0.45 to 10 K are shown in Fig. 2B, where the value (95 *μ*_B_) at 0.45 K and 7 T is slightly lower than the saturation value for eight Gd^3+^ and eight Fe^3+^ ions (expected for 8 × (7/2 + 5/2) × 2 = 96 *μ*_B_), likely due to residual thermal effects.

The magnetic susceptibility and magnetization data were analysed using QMC simulations based on the stochastic series expansion (SSE) method, as implemented in the ALPS (Algorithms and Libraries for Physics Simulations) project [35]. Specifically, the Directed Loop algorithm [36] was employed to efficiently update the loop configurations in the path integral representation, which is particularly suitable for the unfrustrated bipartite lattice of the {Fe_8_Gd_8_} ring. Previous experimental observations on the isostructural {Sc_8_Gd_8_} analogue confirmed that the Gd–Gd interaction is vanishingly small *J*_Gd–Gd_ = −0.013 K, which should be negligible compared to the dominant Fe–Gd exchange. Thus, in our model we assume the magnetic interactions between next-nearest-neighbour Gd^3+^ ions (*J*_Gd–Gd_) as zero, which also is consistent with theoretical treatments of similar {Fe_10_Gd_10_} cyclic clusters reported previously [38]. To determine the exchange coupling constants (*J*_Fe–Fe_, *J*_Fe–Gd_) and the Lande *g*-factor scaling accurately, we developed an automated fitting procedure. The procedure integrates the QMC engine with the Nelder–Mead simplex algorithm, provided by the SciPy package, to minimize the root-mean-square error between the experimental and simulated magnetization isotherms. Through iterative fitting to the experimental magnetization data, the best-fit parameters were obtained as *J*_Fe–Gd_ = 4.81(5) K and *J*_Fe–Fe_ = −0.13(4) K. Magnetization curves simulated with these two *J* values (Fig. 2) are in excellent agreement with experiment. Note that the large and positive *J*_Fe–Gd_ signifies the exchange-coupling between Fe^3+^ and Gd^3+^ ions is ferromagnetic and dominant within the molecule. We postulate that this is the primary driver to cause such an evident MS effect (see below). While ferromagnetic Fe–Gd coupling has been reported in other cyclic systems, such as the {Fe_10_Gd_10_} wheel with a weaker *J*_Fe–Gd_ ≈ 1.0 K (0.7 cm^−1^) [38], the coupling strength in our {Fe_8_Gd_8_} analogue of 4.81(5) K (3.34 cm^−1^) is significantly larger. We attribute this enhanced coupling in {Fe_8_Gd_8_} to the specific coordination environment of the mdea^2−^ ligands, which leads to shorter Fe–Gd intermetallic distances (3.39–3.48 Å) and more favourable alkoxo-bridging angles that maximize the ferromagnetic superexchange pathway. Conversely, the Fe–Fe interaction is negligible (*J*_Fe–Fe_ = −0.13(4) K). This is structurally consistent with the alternating topology of the ring, where Fe^3+^ ions act as next-nearest neighbours separated by Gd^3+^ centres. The absence of direct bridging ligands and the large separation distance between iron centres preclude significant magnetic communication. This is in distinct contrast to the antiferromagnetic Fe–Gd interactions of *J*_Fe–Gd_ = −5.75 K (−4.0 cm^−1^) observed in other high-nuclearity clusters such as the {Gd_12_Fe_14_} system [34], where the antiparallel alignment reduces the total ground-state spin. In distinct contrast, our QMC simulations and magnetization data confirm that the robust ferromagnetic coupling (*J*_Fe–Gd_ = 4.81(5) K) might lead to a high-spin ground state and thereby a giant molecular magnetic moment. The latter becomes a dominant source of magnetoelastic response to the external field.

**Figure 3.**
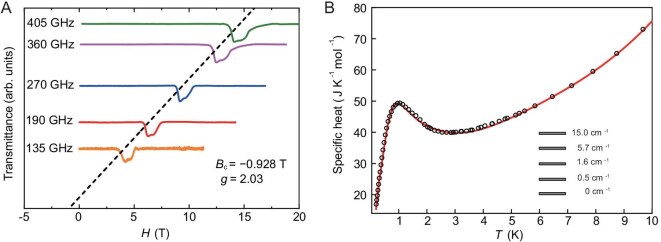
(A) Selected HF-EPR spectra at 4.2 K for {Fe_8_Gd_8_} system. The spectra are offset in a linear scale of the frequency. (B) Temperature dependence of the specific heat measured from 0.2 to 10 K.

HF-EPR measurements (135–405 GHz, 4.2 K) reveal an isotropic *g* value of 2.03(1) and a small zero-field splitting of 0.8 cm^−1^, confirming the presence of minimal single-ion anisotropy (Fig. 3A) [23,25]. Heat capacity measurements over 0.2–10 K show only a broad Schottky anomaly near 1 K, with no λ-type peaks, excluding long-range magnetic ordering. Together, these results rule out significant crystal-field contributions to the MS effect and strongly support an exchange-coupling striction (ECS) origin (Fig. 3B) [25,37,39].

### Magnetostriction measurements and mechanism

**Figure 4.**
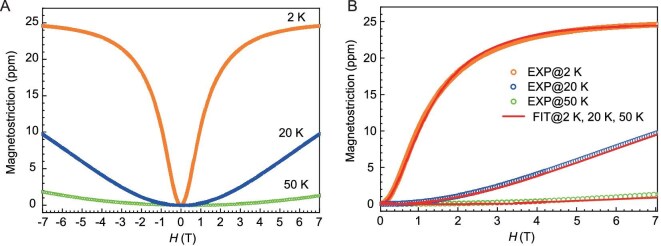
Magnetostriction in {Fe_8_Gd_8_} single crystal: experimental data (0 → 7 T → 0 → −7 T → 0) (A) vs. ECS model (B). Magnetostriction as a function of applied magnetic field for an {Fe_8_Gd_8_} wheel-shaped single crystal, measured at 2 K (orange hollow circles), 20 K (blue hollow circles), and 50 K (green hollow circles). Theoretical magnetostriction curves (solid lines) calculated based on a predominantly ECS contribution (${\epsilon }^{{\mathrm{ECS}}}$) to the strain.

Magnetostriction measurements were performed on a large single crystal of {Fe_8_Gd_8_} using a capacitive dilatometer (Dilatometer Option, Quantum Design) integrated into a physical property measurement system (PPMS). The sample had a wedge-shape geometry suitable for the capacitive dilatometer measurements. The crystal was mounted in a fused silica cell, where the dimensional changes were detected as capacitance changes between the capacitor plates. This technique offers a high resolution (<10 pm) and avoids the mechanical constraints typically imposed by the glue in strain-gauge methods. The characteristic features of Joule magnetostriction are illustrated in Fig. 4A. At 2 K, a visible longitudinal magnetostrictive expansion was observed when the magnetic field was applied along the [100] direction of the cubic crystal plane, while a transverse contraction in the orthogonal direction (Fig. S9). The saturated value reached ∼50 ppm above 7 T. With increasing temperature, the magnetostriction expansions weaken, where only 1 ppm expansion is left for the saturated value. Although the {Fe_8_Gd_8_} crystal possesses macroscopic cubic symmetry in the zero field, the application of an external magnetic field defines a unique axis, thereby breaking the symmetry and generating induced anisotropy. This leads to linear magnetostriction (Joule magnetostriction), characterized by elongation along the field direction and contraction in the perpendicular direction, rather than isotropic volume magnetostriction. This observation aligns with the volume conservation rule, where longitudinal expansion is compensated by transverse contraction [13,14]. Remarkably, the observed saturation magnetostriction (${\lambda }_{\mathrm{s}}$ = 50 ppm) is substantial for a molecular crystal. For context, this value is significantly superior to that of polycrystalline iron (${\lambda }_{\mathrm{s}}$ = 21 ppm) and comparable to polycrystalline nickel (${\lambda }_{\mathrm{s}}$ = −46 ppm). Although it is lower than giant magnetostrictive alloys such as Terfenol-D (${\lambda }_{\mathrm{s}}$ = 2000 ppm) [42], the fact that a soft molecular lattice driven predominantly by intramolecular exchange, without conduction electrons, can achieve metal-level magnetostriction marks a significant conceptual advance.

To provide definitive experimental validation for this exchange-driven mechanism, we measured the magnetostriction of the isostructural ‘magnetic background’ compounds, {Fe_8_Y_8_} and {Sc_8_Gd_8_}, under identical cryogenic conditions. In these diamagnetically substituted analogues, the dominant ferromagnetic Fe–Gd coupling is severed. Strikingly, the macroscopic magnetostriction completely vanishes in both comparative systems (Figs S11, S12). This unambiguous null result explicitly rules out generic background lattice striction and confirms that the giant macroscopic strain in {Fe_8_Gd_8_} is specifically activated by the strong intramolecular Fe–Gd exchange coupling.

According to the unified phenomenological theory developed by the Callens [20], magnetostriction arises from two distinct origins: (i) single-ion crystal field anisotropy and (ii) two-ion exchange interactions. For pure spin states such as Fe^3+^ (3d^5^, *L* = 0) and Gd^3+^ (4f^7^, *L* = 0), the single-ion Stevens operators are strongly suppressed [44], rendering the single-ion magnetostriction negligible. This absence of single-ion contribution in Gd-based systems has been previously utilized to isolate exchange-driven magnetoelastic properties in extended solids [45]. Furthermore, our system lacks the orbital momentum required for the classical single-ion mechanism which typically scales with the generalized Langevin function ${\hat{I}}_{l + 1/2}( m )$ [19]. Therefore, the observed giant magnetostriction is a single-molecule behaviour, which is internally driven by the modulation of the two-ion exchange energy, that is, the ECS mechanism. Although recent studies on cyclic Fe–Gd clusters have suggested that single-ion anisotropy can be non-negligible in analysing magnetic thermodynamics and relaxation [45], we confirm its contribution is minimal in our system based on two key observations. Crucially, our HF-EPR measurements provide a stringent quantitative bound for the residual single-ion anisotropy in this system. The data reveal a nearly isotropic *g*-factor (2.03(1) and a negligible zero-field splitting (*D* ≈ 0.8(1) cm^−1^), which is significantly smaller than the dominant ferromagnetic exchange interaction (*J*_Fe–Gd_ = 4.81(5) K). Therefore, rather than categorically eliminating crystal-field effects, we quantitatively establish that they are suppressed sufficiently to be treated as a minor perturbation relative to the exchange-striction mechanism. The highly symmetric cubic space group ($Pn\bar{3}n$) of the {Fe_8_Gd_8_} lattice naturally minimizes the macroscopic manifestation of local anisotropy, distinct from lower-symmetry analogues. Therefore, we conclude that the observed magnetostriction is an amplified manifestation of a single-molecule striction, originating primarily from the modulation of intramolecular two-ion exchange energy, that is, the ECS mechanism.

To model the magnetostriction, we developed a custom computational workflow combining QMC simulations with a mean-field approximation (MFA) evaluation (Fig. S10). To maintain theoretical rigor, we first utilized the MFA to analytically bound the strain-dependent crystal field derivative. Consistent with our HF-EPR observations, this evaluation confirmed that the single-ion crystal field striction term (${\epsilon }^{{\mathrm{CF}}}$) is quantitatively negligible. Consequently, we reduced our primary fitting model to focus primarily on the modulation of the two-ion exchange energy, adopting the formalism derived by the Callens for two-ion ECS mechanism (${\epsilon }^{{\mathrm{ECS}}}$), where the magnetostriction coefficients are directly proportional to the isotropic spin–spin correlation functions [19]. Specifically, the lattice strain $\epsilon $ minimizes the total free energy by coupling to the scalar invariant $\sum \langle {\hat{S}}_{{\mathrm{Fe}}} \cdot {\hat{S}}_{{\mathrm{Gd}}} \rangle $. Thus, we modelled the magnetostriction as a linear combination of the thermal averages of the local spin correlations calculated by QMC. The formulation relates the magnetostriction directly to the two-ion spin correlation functions ${\mathrm{ \langle }}{\hat{S}}_{{\mathrm{Fe}}} \cdot {\hat{S}}_{{\mathrm{Gd}}}{\mathrm{ \rangle }}$, consistent with the magnetoelastic Hamiltonian derived for systems dominated by exchange interactions. This approach aligns with the McPhase simulation framework established for intermetallic compounds and spin-ice systems, where the total strain is decoupled into crystal field and exchange contributions, with the latter being proportional to the derivative of the exchange integral with respect to strain [43,46,47]. Given the isotropic nature of Fe^3+^ and Gd^3+^ in our cubic lattice and the minimal zero-field splitting, we treat the crystal field term (${\epsilon }^{{\mathrm{CF}}}$) as negligible and focus our model on the exchange term (${\epsilon }^{{\mathrm{ECS}}}$). Due to the prohibitive Hilbert space size of {Fe_8_Gd_8_} ring (∼10^13^), exact diagonalization is not feasible. We employed the SSE algorithm provided by the ALPS libraries to calculate the thermal averages of the local spin correlations ${\mathrm{ \langle }}{\hat{S}}_{{\mathrm{Fe}}} \cdot {\hat{S}}_{{\mathrm{Gd}}}{\mathrm{ \rangle }}$ and ${\mathrm{ \langle }}{\hat{S}}_{{\mathrm{Fe}}} \cdot {\hat{S}}_{{\mathrm{Fe}}}{\mathrm{ \rangle }}$. The calculations were executed on a parallel computing architecture. For each experimental magnetic field point, independent QMC runs were performed with 10^5^ thermalization steps and 10^6^ measurement steps to ensure statistical convergence.


(1) \begin{eqnarray*}
&&{\mathrm{\hat{H}}}_{{\mathrm{ECS}}}^{\mathrm{i}}\left( \epsilon \right){\mathrm{\ = \ -}}\frac{1}{2}\mathop \sum \limits_{{\mathrm{j}}\epsilon {\mathrm{FeGd}}\left( {\mathrm{i}} \right)} {{\mathrm{J}}}_{{\mathrm{Fe \\!-\\! Gd}}}\left( \epsilon \right){\mathrm{ \langle }}{{\mathrm{\hat{S}}}}_{{\mathrm{Fe}}} \cdot {{\mathrm{\hat{S}}}}_{{\mathrm{Gd}}}{\mathrm{ \rangle }} \\
&&\quad -\,\frac{1}{2}\mathop \sum \limits_{{\mathrm{j}}\epsilon {\mathrm{FeFe}}\left( {\mathrm{i}} \right)} {{\mathrm{J}}}_{{\mathrm{Fe \\!-\\! Fe}}}\left( \epsilon \right){\mathrm{ \langle }}{{\mathrm{\hat{S}}}}_{{\mathrm{Fe}}} \cdot {{\mathrm{\hat{S}}}}_{{\mathrm{Fe}}}{\mathrm{ \rangle }}{\mathrm{.}}
\end{eqnarray*}

To ensure the uniqueness of the solution, the exchange coupling constants *J*_Fe–Fe_ and *J*_Fe–Gd_ were fixed to values determined from independent magnetic susceptibility measurements. The resulting simulated magnetostriction curves (Fig. 4B) are in excellent agreement with the experiment, quantitatively verifying that the macroscopic strain is predominantly driven by the modulation of these two-ion exchange mechanism. The excellent agreement between our computational model and experimental data quantitatively verifies that this magnetostriction is intrinsically localized at the single-molecule level, internally governed by the ECS mechanism.

We have observed an impressive Joule magnetostriction in a cubic crystal composed of van der Waals packing of {Fe_8_Gd_8_} molecules up to 50 K. At 2 K, such a magnetostriction can be enhanced to 50 ppm at 7 T. This observation is particularly intriguing when considering the fundamental exchange mechanisms. In metals such as iron or nickel, magnetostriction is driven by strong direct exchange or itinerant electron interactions. In giant magnetostrictive alloys such as Terfenol-D, the performance is often enhanced by the morphotropic phase boundary mechanism [41]. In contrast, the {Fe_8_Gd_8_} system relies on superexchange interactions mediated by oxygen bridges, which are inherently weaker. While the lack of long-range magnetic order excludes magnetic domain effects, the macroscopic strain measured experimentally inherently relies on weak cooperative lattice effects. When the dominant intramolecular exchange-coupling drives the initial deformation of individual {Fe_8_Gd_8_} molecules, these intermolecular elastic interactions cooperatively transmit the local distortions across the crystal. Therefore, the observed giant magnetostriction is a synergistic macroscopic manifestation of an intramolecular magnetic driving force coupled with an intermolecular elastic transmission medium. Since both Fe^3+^ and Gd^3+^ have the quenched orbital angular momentum and the solid is crystallized in the cubic symmetry, the crystal field effect for magnetostriction is negligible, thus, the {Fe_8_Gd_8_} wheel stands as a single-molecule magnetostrictor. Supported by QMC calculation, we further elaborate that this magnetostriction is triggered primarily at the single-molecule level by the ferromagnetic interactions between the Fe^3+^ and Gd^3+^ ions. This is conceptually analogous to the parameter in the Bean–Rodbell theory [48], which describes how the system minimizes its free energy through lattice distortion to enhance favourable magnetic exchange interactions. A similar mechanism has been quantitatively verified in simple molecular solids such as $\beta \hbox{-} {O}_2$, where lattice expansion significantly reduces the antiferromagnetic exchange energy to accommodate the Zeeman energy [49]. In our {Fe_8_Gd_8_} case, the strong Fe–Gd ferromagnetic exchange acts effectively as a mediator of strong spin-lattice coupling at the molecular level, driving lattice deformation in response to the external field. As exchange-coupling is ubiquitous, we expect this new mechanism may inspire a new design for molecule-based magnetostrictive materials.

## MATERIALS AND METHODS

### Synthesis

All reagents and solvents were obtained from commercial suppliers and used without further purification. A mixture of Gd(NO_3_)_3_·5H_2_O (433 mg, 1 mmol), Fe(acac)_3_ (353 mg, 1 mmol), N-methyldiethanolamine (357 mg, 3 mmol), triethylamine (303 mg, 3 mmol), and acetonitrile (8 mL) were sealed in a 10 mL glass vial and then heated at 130°C with autogenous pressure. After 3 days, reddish-brown block crystals were isolated, washed with acetonitrile. Yield: 308 mg, 51.9% based on Gd. For {Fe_8_Gd_8_} complex, elemental analysis (calcd., found): C (28.86, 28.35), H (5.25, 5.49), N (5.02, 4.82). Fourier-transform infrared spectroscopy (FT-IR) (KBr pellets, cm^−1^): 3700–3350 (br, w), 3000 (w), 2958 (w), 2884 (w), 1655 (w), 1557 (s), 1438 (s), 1409 (s), 1341 (w), 1291 (m), 1260 (w), 1203 (w), 1144 (w), 1081 (s), 1033 (m), 997 (m), 932(w), 894 (s), 760 (w), 677 (m), and 654(s).

### X-ray crystallography

Single-crystal X-ray diffraction data for {Fe_8_Gd_8_} complex were collected at 150 K on a Bruker Apex CCD II area-detector diffractometer with graphite-monochromated MoK radiation (*λ* = 0.71 073 Å). Absorption corrections were performed via a multi-scan technique. The structures were solved using the direct method in SHELXTL and refined by full-matrix least-square techniques with the SHELXTL and OLEX2 program. Disordered guest molecules, such as water and acetonitrile, were handled using the SQUEEZE function in the PLATON software. Anisotropic thermal parameters were assigned to all non-hydrogen atoms, while hydrogen atoms were geometrically generated. [CCDC 2310020 contains the supplementary crystallographic data for this paper.]

### Elemental analyses

Elemental analyses of C, H, and N components were carried out using a EUROVECTOR EA3000 elemental analyser.

### Infrared spectra

Infrared spectra for all samples, covering the range of 4000–650 cm^−1^, were obtained using a Thermo Scientific Nicolet 6700 FT-IR spectrophotometer.

### Magnetic properties

Magnetic susceptibility and magnetization measurements were performed between 2 and 300 K using the Quantum Design MPMS superconducting quantum interference device. High-field magnetization measurements were conducted at Tohoku University using a non-destructive pulsed magnet system. Fast pulsed magnetic fields with sweep rates were generated by discharging a 90 kJ capacitor bank. The magnetization data were recorded over a complete sweep cycle using a standard inductive method. To maintain adiabatic conditions and achieve temperatures as low as 0.40 K, the samples were directly immersed in a liquid ^3^He bath utilizing a ^3^He cryostat insert.

### Magnetostriction measurements

High-resolution magnetostriction measurements were carried out using the Dilatometer Option of the Quantum Design PPMS. The single crystal sample (approx. 2.0 mm × 2.0 mm × 1.0 mm) was mounted in a standard fused silica cell (D101-750). The measurement is based on the capacitive dilatometry technique, where the sample’s expansion or contraction is rigidly coupled to the moving plate of a parallel-plate capacitor. The magnetostriction was measured along the [100] and [001] directions by rotating the sample cell orientation with respect to the magnetic field.

### Heat capacity experiment

The heat capacity measurements were conducted using a Quantum DesignPPMS equipped with a dilution refrigerator (DR). The data were collected via the thermal relaxation method and evaluated using the two-tau model. The background heat capacity (addenda), comprising the sample platform, the thermometer, the heater, and the Apiezon N grease was measured independently and subsequently subtracted from the total heat capacity. To ensure rapid thermalization and prevent internal temperature gradients at low temperatures, a small {Fe_8_Gd_8_} crystal weighing 0.3 mg was used for the measurement down to 0.2 K using DR, and a larger crystal weighing 0.7 mg for the measurement above 2 K using the standard cryostat. The datasets of both samples were normalized to molar heat capacity and then combined together. Finally, the non-magnetic lattice (phonon) contribution was determined by measuring a diamagnetic isostructural analogue {Sc_8_Y_8_} following an identical experimental protocol.

### High-frequency/field electron paramagnetic resonance

HF-EPR measurements were conducted using the same high-field pulsed magnet system, employing Gunn oscillators with frequencies spanning from 135 to 405 GHz. The data were recorded in absorption mode.

## Supplementary Material

nwag300_Supplemental_Files
