# Supplementary material for: Single-molecule magnetostrictor: an {Fe8Gd8} cubic crystal exhibits temperature-dependent magnetostriction
Source: Natl Sci Rev. 2026 May 21;13(13):nwag300. doi: 10.1093/nsr/nwag300 (PMC13352436; doi:10.1093/nsr/nwag300)

checkCIF/PLATON report

You have not supplied any structure factors. As a result the full set of tests cannot be run.

THIS REPORT IS FOR GUIDANCE ONLY. IF USED AS PART OF A REVIEW PROCEDURE FOR PUBLICATION, IT SHOULD NOT REPLACE THE EXPERTISE OF AN EXPERIENCED CRYSTALLOGRAPHIC REFEREE.

No syntax errors found.   CIF dictionary   Interpreting this report

Datablock: Fe8Gd8

Bond precision: C-C = 0.0187 A   Wavelength=0.71073

Cell:	a=39.872(4)	b=39.872(4)	c=39.872(4)
	alpha=90	beta=90	gamma=90
Temperature:	100 K		

	Calculated	Reported
Volume	63388(19)	63389(10)
Space group	P n -3 n	Pn-3n
Hall group	-P 4a 2bc 3	?
Moiety formula	C112 H224 Fe8 Gd8 N16 O64, C2 H3 N [+ solvent]	?
Sum formula	C114 H227 Fe8 Gd8 N17 O64 [+ solvent]	C114 H227 Fe8 Gd8 N17 O64
Mr	4564.94	4745.01
Dx,g cm-3	1.435	1.435
Z	12	12
Mu (mm-1)	3.070	3.070
F000	27144.0	27144.0
F000'	27172.40	
h,k,lmax	47,47,47	47,47,47
Nref	9358	9352
Tmin,Tmax	0.431,0.641	0.431,0.641
Tmin'	0.398	

Correction method= # Reported T Limits: Tmin=0.431 Tmax=0.641
AbsCorr = MULTI-SCAN

Data completeness= 0.999   Theta(max)= 25.010

R(reflections)= 0.0938(8539)   wR2(reflections)= 0.2064(9352)

S = 1.183   Npar= 479

The following ALERTS were generated. Each ALERT has the format
test-name_ALERT_alert-type_alert-level.
Click on the hyperlinks for more details of the test.

Alert level B

PLAT990_ALERT_1_B Deprecated .res/.hkl Input Style SQUEEZE Job ... ! Note

---

Alert level C

ABSTY02_ALERT_1_C An _exptl_absorpt_correction_type has been given without
a literature citation. This should be contained in the
_exptl_absorpt_process_details field.
Absorption correction given as Multi-scan
CHEMW01_ALERT_1_C The ratio of given/expected molecular weight as calculated
from the _chemical_formula_sum lies outside
the range 0.99 <> 1.01
Calculated formula weight = 4564.8989
Formula weight given = 4745.0098
CHEMW01_ALERT_1_C The difference between the given and expected weight for
compound is greater 1 mass unit. Check that all hydrogen
atoms have been taken into account.
DENSD01_ALERT_1_C The ratio of the submitted crystal density and that
calculated from the formula is outside the range 0.99 <> 1.01
Crystal density given = 1.435
Calculated crystal density = 1.491
PLAT046_ALERT_1_C Reported Z, MW and D(calc) are Inconsistent .... 1.492 Check
PLAT094_ALERT_2_C Ratio of Maximum / Minimum Residual Density .... 2.18 Report
PLAT213_ALERT_2_C Atom C22 has ADP max/min Ratio ..... 3.3 prolat
PLAT232_ALERT_2_C Hirshfeld Test Diff (M-X) Gd1 --O4 . 5.7 s.u.
PLAT234_ALERT_4_C Large Hirshfeld Difference N4 --C21 . 0.16 Ang.
PLAT234_ALERT_4_C Large Hirshfeld Difference C20 --C21 . 0.16 Ang.
PLAT241_ALERT_2_C High 'MainMol' Ueq as Compared to Neighbors of C7 Check
PLAT241_ALERT_2_C High 'MainMol' Ueq as Compared to Neighbors of C9 Check
PLAT241_ALERT_2_C High 'MainMol' Ueq as Compared to Neighbors of C21 Check
PLAT241_ALERT_2_C High 'MainMol' Ueq as Compared to Neighbors of C23 Check
PLAT242_ALERT_2_C Low 'MainMol' Ueq as Compared to Neighbors of N2 Check
PLAT242_ALERT_2_C Low 'MainMol' Ueq as Compared to Neighbors of N4 Check
PLAT243_ALERT_4_C High 'Solvent' Ueq as Compared to Neighbors of C2S Check
PLAT260_ALERT_2_C Large Average Ueq of Residue Including N1S 0.276 Check
PLAT342_ALERT_3_C Low Bond Precision on C-C Bonds .... 0.01869 Ang.
PLAT360_ALERT_2_C Short C(sp3)-C(sp3) Bond C9 - C10 . 1.42 Ang.
PLAT360_ALERT_2_C Short C(sp3)-C(sp3) Bond C13 - C14 . 1.37 Ang.
PLAT360_ALERT_2_C Short C(sp3)-C(sp3) Bond C23 - C24 . 1.38 Ang.
PLAT413_ALERT_2_C Short Inter XH3 .. XHn H27A ..H27C . 2.12 Ang.
1/2+y,1-x,-z = 40_665 Check

Alert level G

PLAT002_ALERT_2_G Number of Distance or Angle Restraints on AtSite 3 Note
PLAT003_ALERT_2_G Number of Uiso or U(i,j) Restrained non-H Atoms 10 Report

```
PLAT005_ALERT_5_G No Embedded Refinement Details Found  in the CIF     Please Do !
PLAT066_ALERT_1_G Predicted and Reported Tmin&Tmax Range Identical          ? Check
PLAT083_ALERT_2_G SHELXL Second Parameter in WGHT  Unusually Large    2915.31 Why ?
PLAT152_ALERT_1_G The Supplied and Calc. Volume s.u. Differ by ...          9 Units
PLAT300_ALERT_4_G Atom Site Occupancy of H1S1       Constrained at       0.25 Check
PLAT300_ALERT_4_G Atom Site Occupancy of H1S2       Constrained at       0.25 Check
PLAT300_ALERT_4_G Atom Site Occupancy of H1S3       Constrained at       0.25 Check
PLAT380_ALERT_4_G Incorrectly? Oriented X(sp2)-Methyl Moiety .....        C12 Check
PLAT380_ALERT_4_G Incorrectly? Oriented X(sp2)-Methyl Moiety .....        C25 Check
PLAT606_ALERT_4_G Solvent Accessible VOID(S) in Structure ........          ! Info
PLAT608_ALERT_4_G ADDSYM Test Skipped (Too Time-consuming) .......          ! Info
PLAT720_ALERT_4_G Number of Unusual/Non-Standard Labels ..........          3 Note
              H1S1    H1S2    H1S3
PLAT764_ALERT_4_G Overcomplete CIF Bond List Detected (Rep/Expd) .       1.14 Ratio
PLAT794_ALERT_5_G Tentative Bond Valency for Fe1       (III)     .       2.99 Info
PLAT794_ALERT_5_G Tentative Bond Valency for Fe2       (III)     .       2.95 Info
PLAT860_ALERT_3_G Number of Least-Squares Restraints .............         65 Note
PLAT869_ALERT_4_G ALERTS Related to the Use of SQUEEZE  Suppressed          ! Info
PLAT899_ALERT_4_G SHELXL-97  is Outdated   and Succeeded by SHELXL     2019/3 Note
```

---

0 **ALERT level A** = Most likely a serious problem - resolve or explain
1 **ALERT level B** = A potentially serious problem, consider carefully
23 **ALERT level C** = Check. Ensure it is not caused by an omission or oversight
20 **ALERT level G** = General information/check it is not something unexpected

8 ALERT type 1 CIF construction/syntax error, inconsistent or missing data
17 ALERT type 2 Indicator that the structure model may be wrong or deficient
2 ALERT type 3 Indicator that the structure quality may be low
14 ALERT type 4 Improvement, methodology, query or suggestion
3 ALERT type 5 Informative message, check

It is advisable to attempt to resolve as many as possible of the alerts in all categories. Often the minor alerts point to easily fixed oversights, errors and omissions in your CIF or refinement strategy, so attention to these fine details can be worthwhile. In order to resolve some of the more serious problems it may be necessary to carry out additional measurements or structure refinements. However, the purpose of your study may justify the reported deviations and the more serious of these should normally be commented upon in the discussion or experimental section of a paper or in the "special_details" fields of the CIF. checkCIF was carefully designed to identify outliers and unusual parameters, but every test has its limitations and alerts that are not important in a particular case may appear. Conversely, the absence of alerts does not guarantee there are no aspects of the results needing attention. It is up to the individual to critically assess their own results and, if necessary, seek expert advice.

Publication of your CIF in IUCr journals

A basic structural check has been run on your CIF. These basic checks will be run on all CIFs submitted for publication in IUCr journals (*Acta Crystallographica*, *Journal of Applied Crystallography*, *Journal of Synchrotron Radiation*); however, if you intend to submit to *Acta Crystallographica Section C* or *E* or *IUCrData*, you should make sure that full publication checks are run on the final version of your CIF prior to submission.

Publication of your CIF in other journals

Please refer to the *Notes for Authors* of the relevant journal for any special instructions relating to CIF submission.

PLATON version of 22/08/2024; check.def file version of 21/08/2024

Datablock Fe8Gd8 - ellipsoid plot

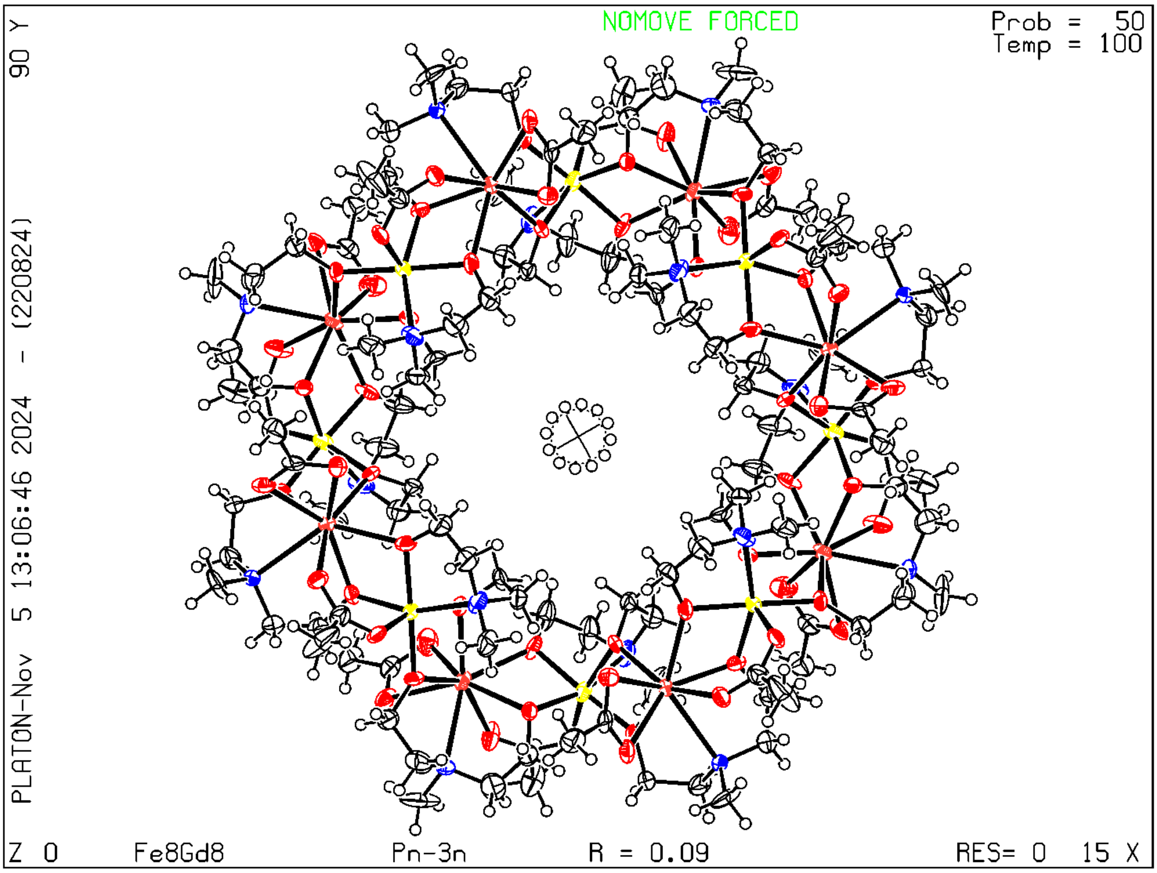

Supplement: nwag300_Supplemental_Files [file nwag300_supplemental_files.zip › CheckCIF_File.pdf]
