# Supplementary material for: Single-molecule magnetostrictor: an {Fe8Gd8} cubic crystal exhibits temperature-dependent magnetostriction
Source: Natl Sci Rev. 2026 May 21;13(13):nwag300. doi: 10.1093/nsr/nwag300 (PMC13352436; doi:10.1093/nsr/nwag300)
Supplement: nwag300_Supplemental_Files [file nwag300_supplemental_files.zip › NSR_MS-2026-274.R2_SI.pdf]

Supporting Information

Single-molecule magnetostrictor: an {Fe_8Gd_8} cubic crystal exhibits temperature-dependent magnetostriction

Dong-Yang Li[1,†], Lei Qin[1,†], Yuan-Qi Zhai[1,*], Jia-Shu Sun[1], Xia-Li Ding[2], Jin-Tao Lu[3,4], Ismael Francisco Díaz-Ortega[5,6], Hiroyuki Nojiri[5], Zhendong Fu[7,*] and Yan-Zhen Zheng[1,*]

[1]Frontier Institute of Science and Technology, Interdisciplinary Research Center of Frontier Science and Technology, State Key Laboratory of Electrical Insulation and Power Equipment, Xi'an Key Laboratory of Electronic Devices and Materials Chemistry, Xi'an Jiaotong University, Xi'an 710054, China;
[2]Shaanxi Science and Technology Holding Institute, Xi'an 710016, China;
[3]Key Laboratory of the Ministry of Education and International Center for Dielectric Research, School of Electronic Science and Engineering, Xi'an Jiaotong University, Xi'an 710049, China;
[4]Xi'an SIMOLDE Science and Technology Co., Ltd, Xi'an 710049, China;
[5]Institute for Materials Research (IMR), Tohoku University, Sendai 980-8577, Japan;
[6]Departamento de Química y Física-CIESOL, Universidad de Almería, Ctra. Sacramento s/n, 04120 Almería, Spain;
[7]Songshan Lake Materials Laboratory, Dongguan 523808, China

* Corresponding authors. E-mails: zhaiyuanqi@xjtu.edu.cn; zdfu@pku.edu.cn; zheng.yanzhen@xjtu.edu.cn.
[†]Equally contributed to this work.

Molecular Hirshfeld surfaces

Fig. S4 illustrates the maps of d_{norm}, d_i, d_e, shape index, and curvedness on molecular HSs. Here, d_e indicates the distance from a surface point to the nearest internal atom, while d_i denotes the distance to the nearest external atom, also considering the van der Waals ratio of the atom. In the 2D fingerprint plots, as seen in Fig. S5, on the d_{norm} surface, adjacent white-blue areas highlight weak interaction between molecules. These interactions are evident on Hirshfeld surfaces and in the fingerprint plots. These plots can be decomposed to emphasize specific atom pair contacts, allowing for the separation of different interaction types that overlap in the full fingerprint. Most of the total HSs comprises H...H/H...H accounting for 67.8%. Other significant interactions include H...O/H...O (6.0%), N...H/N...H (1.5%) and C...H/C...H (1.2%) contacts for the title molecule. These intermolecular contacts, although weak, play a significant role in stabilizing the molecular packing. Therefore, while the primary origin of the MS effect appears to be intramolecular, these weak cooperative effects may still contribute to the overall system stability, albeit to a lesser extent.

Pulsed-Field Magnetization Measurements

High-field magnetization measurements under adiabatic conditions were performed using a non-destructive pulsed magnet system. To strictly mitigate adiabatic heating and ensure complete thermal equilibrium during the rapid magnetic field pulses, the sample was directly immersed in a liquid ^{3}He bath. The extremely small dimensions of the crystallites provided a highly favourable surface-to-volume ratio, facilitating near-instantaneous heat exchange with the cryogenic liquid. This setup effectively suppressed any non-equilibrium thermal bottlenecks, ensuring the sample temperature remained stable at 0.45 K throughout the pulse sequences.

The pulsed magnetic field was generated by discharging a capacitor bank. The temporal profile of the magnetic pulse was carefully monitored. The instantaneous sweep rates dH/dt were extracted directly from the magnetic field waveform. During the up-sweep phase (increasing field), the sweep rate was approximately 15535 T/s at 1 T and 12154 T/s at 5 T. During the down-sweep phase (decreasing field), the rates were approximately 8083 T/s at 1 T and 8278 T/s at 5 T. The magnetization data were recorded over complete, continuous magnetic field sweep cycles (0 → 7 T → 0 → -7 T→ 0). The signal was obtained by numerically integrating the induced voltage from the pick-up coils. Careful comparison between the up-sweep and down-sweep curves revealed only a remarkably tiny hysteresis, confirming that the magnetization process is an instantaneous thermodynamic equilibrium response and verifying that the extracted parameters are completely free from non-equilibrium or dynamic bias effects. For data analysis, the *M-H* curves were averaged between the up and down sweeps.

High-frequency/field electron paramagnetic resonance

High frequency/field electron paramagnetic resonance (HF-EPR) spectra were recorded at 4.2 K, using multi-frequency of 135−405 GHz in pulsed magnetic fields

up to 20 T (Fig. 3A). The absorption peaks shift to higher fields as the frequency increases, corresponding to the transitions between the zero-field split M_s sublevels of the high-spin ground state. Clearly the linear fit of the data reveals a negative bias field, deviating from the typical Zeeman effect, resulting in an estimated g_{iso} around 2.03(1) and the level-crossing field B_c at −0.928 T. The zero-field gap of 24 GHz (0.8(1) cm^{-1}) corresponds to the exchange interaction between Gd^{3+} and Fe^{3+} ions, quantitatively supporting the intramolecular ECS mechanism that drives this single-molecule behaviour.

Heat capacity

The molar specific heat measurement of $\{Fe_8Gd_8\}$ crystal was performed from 0.2 to 10 K in zero magnetic field (**Fig. 3B**). The Schottky anomaly at about 1 K provides clear evidence of low-lying magnetic excitations. The magnetic specific heat C_{mag} is fitted using the Schottky model for a multi-level system, which can be described by

$$C_{mag} = \frac{R}{k_B^2 T^2}\left\{\frac{\sum_i \varepsilon_i^2 \exp\left(-\frac{\varepsilon_i}{k_B T}\right)}{\sum_i \exp\left(-\frac{\varepsilon_i}{k_B T}\right)} - \left[\frac{\sum_i \varepsilon_i \exp\left(-\frac{\varepsilon_i}{k_B T}\right)}{\sum_i \exp\left(-\frac{\varepsilon_i}{k_B T}\right)}\right]^2\right\}, \quad (1)$$

where ε_i is the energy of spin level i. Four effective energy gaps (Δ_1–Δ_4, defined with $\Delta_i = \varepsilon_i - \varepsilon_0$) as shown in **Fig. 3B** are resolved from the fit to heat capacity data. Considering the inherent thermal broadening of macroscopic specific heat measurements, the first energy gap $\Delta_1 = 0.5(1)$ cm^{-1} extracted from the hump around 1 K is highly consistent with the 0.8 cm^{-1} energy gap precisely resolved by HF-EPR. Both values fall within the <1 cm^{-1} range, making them reasonably close. They originate from the ZFS between the quantum levels $m_s = 0$ and $m_s = \pm 1$ within the high-spin ground state manifold. Furthermore, the larger phenomenological energy gaps ($\Delta_2 = 1.6$ cm^{-1}, $\Delta_3 = 5.7$ cm^{-1} and $\Delta_4 = 15.0$ cm^{-1}) are required to reproduce the broad and continuous signal of magnetic specific heat above 2 K. Rather than representing isolated single transitions, these values effectively reflect the progressive thermal population of higher-order m_s sublevels of the ground state and/or low-lying excited spin multiplets, which overlap to form a broad thermodynamic response.

Quantum Monte Carlo computational details

1. Structural characterization and magnetic coupling model of the Fe_8Gd_8 cluster

The Fe_8Gd_8 is a high symmetry, cyclic heterometallic cluster. The magnetic properties are analysed using the isotropic Heisenberg Hamiltonian, augmented by the Zeeman term, as defined below:

$$\hat{H} = -\sum_{i<j} J_{ij} \hat{s}_i \cdot \hat{s}_j + g_s \mu_B \sum_i H \cdot \hat{s}_i \quad (2)$$

where S_i and S_j are the spin operators for the interacting metal ions, J_{ij} is the exchange coupling constant, and the second term accounts for the coupling to the external magnetic field H.

The complete Hamiltonian used in the program is given below:

$$\hat{H} = -J_{Fe\text{-}Gd}(\hat{\vec{s}}_{Fe1}\hat{\vec{s}}_{Gd1} + \hat{\vec{s}}_{Fe2}\hat{\vec{s}}_{Gd1} + \hat{\vec{s}}_{Fe2}\hat{\vec{s}}_{Gd2} + \hat{\vec{s}}_{Fe3}\hat{\vec{s}}_{Gd2} + \hat{\vec{s}}_{Fe3}\hat{\vec{s}}_{Gd3} + \hat{\vec{s}}_{Fe4}\hat{\vec{s}}_{Gd3} + \hat{\vec{s}}_{Fe4}\hat{\vec{s}}_{Gd4}$$
$$+ \hat{\vec{s}}_{Fe5}\hat{\vec{s}}_{Gd4} + \hat{\vec{s}}_{Fe5}\hat{\vec{s}}_{Gd5} + \hat{\vec{s}}_{Fe6}\hat{\vec{s}}_{Gd5} + \hat{\vec{s}}_{Fe6}\hat{\vec{s}}_{Gd6} + \hat{\vec{s}}_{Fe7}\hat{\vec{s}}_{Gd6} + \hat{\vec{s}}_{Fe7}\hat{\vec{s}}_{Gd7} + \hat{\vec{s}}_{Fe8}\hat{\vec{s}}_{Gd7} + \hat{\vec{s}}_{Fe8}\hat{\vec{s}}_{Gd8} + \hat{\vec{s}}_{Fe1}\hat{\vec{s}}_{Gd8})$$
$$- J_{Fe\text{-}Fe}(\hat{\vec{s}}_{Fe1}\hat{\vec{s}}_{Fe2} + \hat{\vec{s}}_{Fe2}\hat{\vec{s}}_{Fe3} + \hat{\vec{s}}_{Fe3}\hat{\vec{s}}_{Fe4} + \hat{\vec{s}}_{Fe4}\hat{\vec{s}}_{Fe5} + \hat{\vec{s}}_{Fe5}\hat{\vec{s}}_{Fe6} + \hat{\vec{s}}_{Fe6}\hat{\vec{s}}_{Fe7} + \hat{\vec{s}}_{Fe7}\hat{\vec{s}}_{Fe8} + \hat{\vec{s}}_{Fe8}\hat{\vec{s}}_{Fe1}) \quad (3)$$

The metal ions possess high spin quantum numbers: $S_{Fe} = 5/2$ for Fe^{3+} and $S_{Gd} = 7/2$ for the isotropic Gd^{3+} ions. Due to the cubic space group ($Pn\bar{3}n$), the exchange interactions are simplified into two parameters:

$J_{Fe\text{-}Gd}$: $Fe^{3+} - Gd^{3+}$ exchange coupling

$J_{Fe\text{-}Fe}$: $Fe^{3+} - Fe^{3+}$ exchange coupling

2. Quantum Monte Carlo (QMC) simulation method

The total spin dimension of the Fe_8Gd_8 cluster is $D = (2\hat{S}_{Fe} + 1)^8 \times (2\hat{S}_{Gd} + 1)^8 = 6^8 \times 8^8 \approx 2.82\times10^{13}$, precluding the use of full matrix diagonalization. Consequently, we employed the highly efficient Stochastic Series Expansion(SSE) - Quantum Monte Carlo method. The SSE-QMC simulations were performed using the dirloop_sse solver within the ALPS (Algorithms and Libraries for Physics simulations) package. The SSE algorithm is well suited for large interacting spin systems, as is computational complexity scaled weakly with the number of spins and is independent of the maximum local spin quantum number(S).

3. Parameter optimization using differential evolution

To determine the optimal set of exchange parameter constants that can reproduce the experimental magnetic data, we utilized a robust global optimization approach coupling the QMC simulations with the Differential Evolution (DE) algorithm implemented via SciPy.

The theoretical magnetization $M_{theo}(H_i)$ obtained from QMC was compared to the experimental data $M_{exp}(H_i)$ by minimizing the Sum of Squared Residuals(SSR), which serves as the fitness function for the DE algorithm:

$$SSR = \sum_i (M_{theo}(H_i) - M_{exp}(H_i))^2 \quad (4)$$

The search range for all coupling constant was initially set to [−10.0:10.0] K. The DE algorithm iteratively refined the population of ($J_{Fe\text{-}Gd}$ and $J_{Fe\text{-}Fe}$) parameters through mutation and crossover operations over multiple generations until the SSR value converge to a global minimum.

Given that the fitting targets the low-temperature saturation region of the M vs H curve, the effect of Temperature-independent Paramagnetism (TIP) and intermolecular mean-field correction (zJ) are considered to be minor perturbations relative to the dominant intramolecular exchange and Zeeman terms.

Magnetostriction mechanism

The Magnetostrictive effect describes the phenomenon in which a magnetic field induces deformation of the crystal lattice, in our discrete {Fe_8Gd_8} system, its core mechanism is fundamentally rooted in intramolecular magnetoelastic coupling at the single-molecule level. We adopt a linear-approximation framework and introduce the strain tensor ϵ to characterize lattice deformation. A magnetic system can reduce its total energy through lattice deformation, and the reduced energy stems from the dependence of exchange interactions and the crystal field Hamiltonian on the strain ϵ.

Regarding the full Hamiltonian, the Zeeman effect component ($-g_s\mu_B \sum_i H{\cdot}S_i$) does not contribute to the striction energy of the system, as it depends only on the alignment of individual spins with the external field and not on interatomic distances. Since this complex only contains Fe^{3+} and Gd^{3+} ions, the influence of the crystal field is expected to be relatively minor. Therefore, the intramolecular exchange-coupling striction (ECS) model ($\hat{H}_{ECS} = -\frac{1}{2}\sum_{ij,\alpha\beta} \hat{s}_i^{\alpha} T_{\alpha\beta}(\epsilon, i-j)\hat{s}_j^{\beta}$) is considered predominantly responsible for triggering the initial deformation at the single-molecule level. As illustrated in **Fig. S1C**, when the magnetic field increases, the magnetic moments ($\vec{\mu} = -\mu_B g_S \vec{S}$) at each Fe^{3+} and Gd^{3+} site tend to align along the z-axis. This alignment led to the increasing of S_z from $S{\cdot}\cos\theta$ to S, thus the eigenvalue (E_{ECS}) of $\hat{H}_{ECS}$ tends to increase. To stabilize the system energetically, the exchange coupling constant $T_{\alpha\beta}$ will tend to decrease. Since $T_{\alpha\beta}$ is inversely proportional to the distance between each pair of ions (r), this decrease in $T_{\alpha\beta}$ corresponds to an increase in r. The resulting expansion of the lattice leads directly to the magnetostriction effect.

To correlate experimental data with theoretical modelling, we performed mean-field calculations according to the following procedure: First, we construct a single-ion Hamiltonian and diagonalize it. This requires first calculating the initial crystal field parameters B_l^m using a point charge model, then constructing the single-ion Hamiltonian H_{SI}^i under the strain-free condition ($\epsilon = 0$) ($\hat{H}_{SI}^i = \hat{H}_0 + \hat{H}_1 + \hat{H}_{SO} + \hat{H}_{CF}^i + \hat{H}_{Ze}$). Subsequently, we obtain the energy level structures and wave functions based on a further simplified Hamiltonian: $\hat{H} = \hat{H}_{CF} + \hat{H}_{ECS} - \sum_i g_s\mu_B S_i{\cdot}H$. Here, only the crystal field (CF) and exchange-coupling striction (ECS) are considered to explicitly bound their respective contributions to the lattice distortion. The crystal field parameters B_l^m depend on the local environment of the ions, which is affected by the strain ϵ . Therefore, the expression of the crystal field Hamiltonian H_{CF} as a function of ϵ is:

$$\hat{H}_{CF}^i(\epsilon) = \sum_{l=0}^{\infty} \sum_{m=-l}^{l} B_l^m(\epsilon, i)\, O_l^m(i) \tag{5}$$

where $B_l^m(\epsilon, i)$ represents crystal field parameters, $O_l^m(i)$ stands for operator equivalents. Besides the minor crystal field term, the lattice strain also significantly

modulates the intramolecular exchange interactions. The strength of exchange interactions depends on the ion spacing in the lattice, and nearest neighbours are usually considered. Here, we take two types of interactions into account: between two Fe^{3+} ions and between an Fe^{3+} ion and a Gd^{3+} ion. The third possible interaction, the next-nearest-neighbour Gd^{3+}–Gd^{3+} coupling ($J_{Gd–Gd}$), is omitted from the model. Based on our previous experimental observations of the isostructural Sc_8Gd_8 analogue, this interaction is vanishingly small ($J_{Gd–Gd} = -0.013$ K). Because it is more than two orders of magnitude smaller than the dominant $J_{Fe–Gd}$ coupling (4.81(5) K), its inclusion would unnecessarily over-parameterize the model while its contribution to the macroscopic exchange-coupling striction (ϵ^{ECS}) is physically negligible. The strain ϵ causes lattice deformation, thereby changing the distance between Fe^{3+} ions and Gd^{3+} ions and further altering the exchange constant J. We construct exchange interactions using a strain-dependent exchange constant $J(\epsilon)$:

$$\hat{H}^{i}_{\mathrm{ECS}}(\epsilon) = -\frac{1}{2}\sum_{j\epsilon \mathrm{FeGd}(i)} J_{\text{Fe-Gd}}(\epsilon)\vec{J}_{\mathrm{i}}\vec{J}_{\mathrm{j}} - \frac{1}{2}\sum_{j\epsilon \mathrm{FeFe}(i)} J_{\text{Fe-Fe}}(\epsilon)\vec{J}_{i}\vec{J}_{j} \tag{6}$$

Under strong magnetic field conditions ($H_{\mathrm{Ze}} > H_{\mathrm{ECS}}$ and $H_{\mathrm{Ze}} > H_{\mathrm{CF}}$), the magnetic moments $\vec{J}_i$ of ions align parallel to the magnetic field direction. At this time, the magnetic system can reduce energy by weakening unfavourable exchange interactions through lattice deformation. The exchange magnetostrictive effect still exists under low-field conditions.

Specifically, we use the point charge model to estimate the magnetoelastic constants of the crystal field $\left[\frac{\partial B_{\mathrm{l}}^{\mathrm{m}}(\epsilon)}{\partial \epsilon}\right]_{\epsilon=0}$, and then estimate the magnetoelastic constants of exchange interactions $\left[\frac{\partial J(\epsilon)}{\partial \epsilon}\right]_{\epsilon=0}$. From this, we can construct a magnetostrictive strain formula for Fe_8Gd_8 to establish the contribution of its magnetoelastic coupling term. Based on the eigenstates obtained through the above steps, we can calculate the corresponding thermodynamic average values:

$$\epsilon' = -\sum_{s=1}^{S}\left(\sum_{l=0}^{\infty}\sum_{m=-l}^{l}\left[\frac{\partial B_{\mathrm{l}}^{\mathrm{m}}(\epsilon)}{\partial \epsilon}\right]_{\epsilon=0}\langle O_l^m\rangle - \frac{1}{2}\sum_{j\epsilon\text{Fe-Gd}}\left[\frac{\partial J(\epsilon)}{\partial \epsilon}\right]_{\epsilon=0}\left\langle S_{\mathrm{i}}^{\alpha}S_{\mathrm{j}}^{\beta}\right\rangle - \frac{1}{2}\sum_{j\epsilon\text{Fe-Fe}}\left[\frac{\partial J(\epsilon)}{\partial \epsilon}\right]_{\epsilon=0}\left\langle S_{\mathrm{i}}^{\alpha}S_{\mathrm{j}}^{\beta}\right\rangle\right) \tag{7}$$

where $<O_{\mathrm{l}}^{\mathrm{m}}>$ is the thermal average value of the Steven operator $O_{\mathrm{l}}^{\mathrm{m}}$. To model the magnetostriction and clearly delineate our theoretical framework, we established a hybrid computational workflow that precisely maps specific methodologies to physical observables (Fig. S10). The exact exchange coupling constants ($J_{Fe–Gd}$, $J_{Fe–Gd}$) and the thermal averages of the local spin-spin correlation functions ($<\hat{S}_{\mathrm{i}}\cdot\hat{S}_{\mathrm{j}}>$) were computed exclusively via the rigorous Quantum Monte Carlo (QMC) engine. The QMC approach is highly sensitive and essential here, as it fully captures the strong

quantum fluctuations and the exact cyclic topology at low temperatures that a mean-field approach would fail to reproduce. Conversely, the mean-field approximation (MFA) was employed conceptually as an analytical bounding tool to evaluate the strain-dependent crystal field derivative ($\left[\frac{\partial B_l^m(\epsilon)}{\partial \epsilon}\right]_{\epsilon=0}$). Given the $L = 0$ states of the Fe^{3+} and Gd^{3+} ions and the highly symmetric $Pn\bar{3}n$ cubic lattice, this mean-field analysis confirms that the single-ion crystal-field striction term (ϵ^{CF}) is minor. Therefore, rather than neglecting the crystal field entirely, we demonstrate that its contribution is theoretically negligible compared to the exchange derivative, allowing us to model the total strain as being predominantly governed by the QMC-derived exchange-coupling striction (ϵ^{ECS}) components. Although the strain-dependent crystal field term (the first term involving $\left[\frac{\partial B_l^m(\epsilon)}{\partial \epsilon}\right]_{\epsilon=0}$) is formally included in Eq. 7 for theoretical completeness, its magnitude is theoretically bounded and expected to be negligible compared to the exchange-coupling derivatives ($\left[\frac{\partial J(\epsilon)}{\partial \epsilon}\right]_{\epsilon=0}$). This is due to the highly symmetric $Pn\bar{3}n$ space group and the quenched orbital angular momentum of the constituent ions. Therefore, our subsequent mean-field analysis assumes the total strain is predominantly governed by the intramolecular exchange striction components, reaffirming its single-molecule origin.

To determine the optimal set of exchange coupling constants and demonstrate the uniqueness of our model, we progressed from independent fittings to a robust global optimization approach. Initially, independent fits were performed for the magnetization $M(H,T)$ and magnetostriction $\lambda(H,T)$ datasets. The theoretical values obtained from the QMC simulations were compared to the experimental data by minimizing the Sum of Squared Residuals (SSR). Following the confirmation of parameter consistency across independent fits, a constrained global fit was executed using a combined objective function:

$$SSR_{\mathrm{global}} = \omega_1 \sum_i (M_{\mathrm{theo}}(H_{\mathrm{i}}) - M_{\mathrm{exp}}(H_{\mathrm{i}}))^2 + \omega_2 \sum_i (\lambda_{\mathrm{theo}}(H_{\mathrm{j}}) - \lambda_{\mathrm{exp}}(H_{\mathrm{j}}))^2 \quad (8)$$

where ω_1 and ω_2 are statistical weights compensating for the distinct dimensional scales of magnetization and strain. The Differential Evolution (DE) algorithm iteratively refined the population of ($J_{\mathrm{Fe-Gd}}$ and $J_{\mathrm{Fe-Fe}}$) parameters until SSR_{global} converged to a global minimum. To quantify the discriminating power of the fit, the covariance matrix was approximated using the residual bootstrapping technique evaluated at the global minimum. The standard errors extracted from the diagonal elements of the covariance matrix were used to establish the 95% confidence intervals for the exchange parameters, confirming that the solution does not reside in a shallow parameter valley. The final globally optimized parameters are $J_{\mathrm{Fe-Gd}}$ = 4.81(5) K and $J_{\mathrm{Fe-Fe}}$ = – 0.13(4) K.

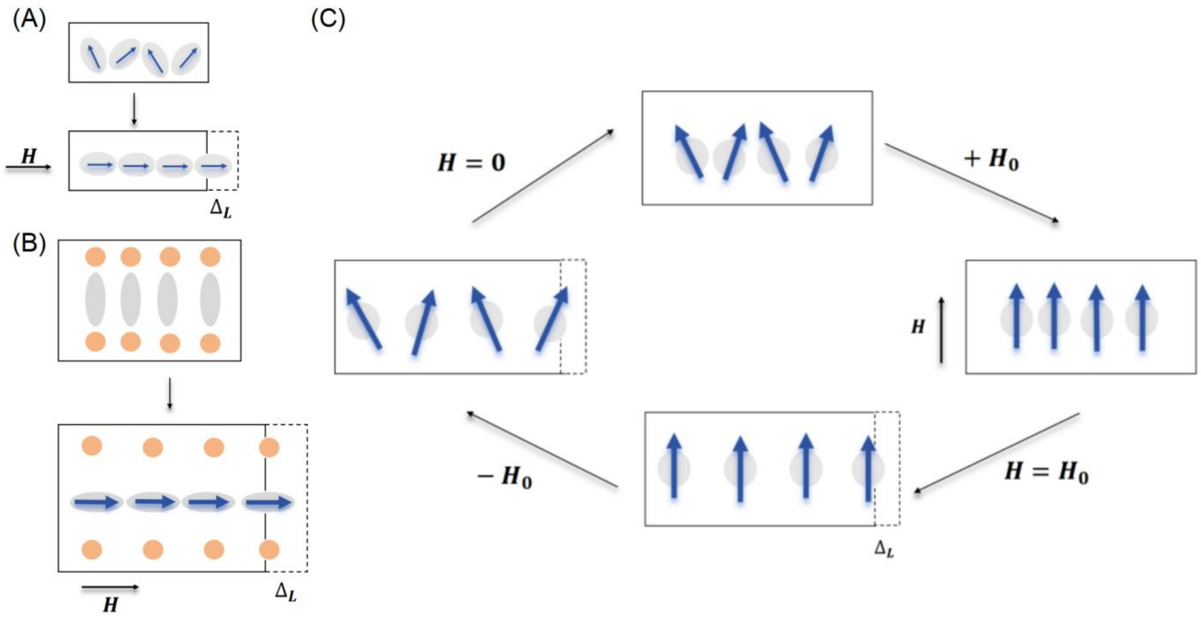

Figure S1. Domain flip (A), crystal field (B) and exchange-coupling striction (C) mechanisms for magnetostriction.

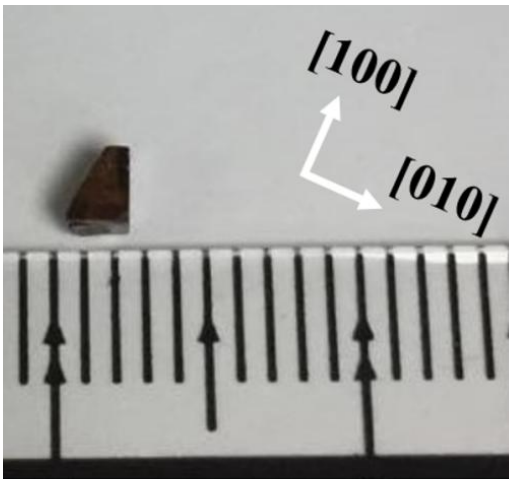

Figure S2. The single-crystal sample of Fe_8Gd_8 used for the magnetostriction measurements.

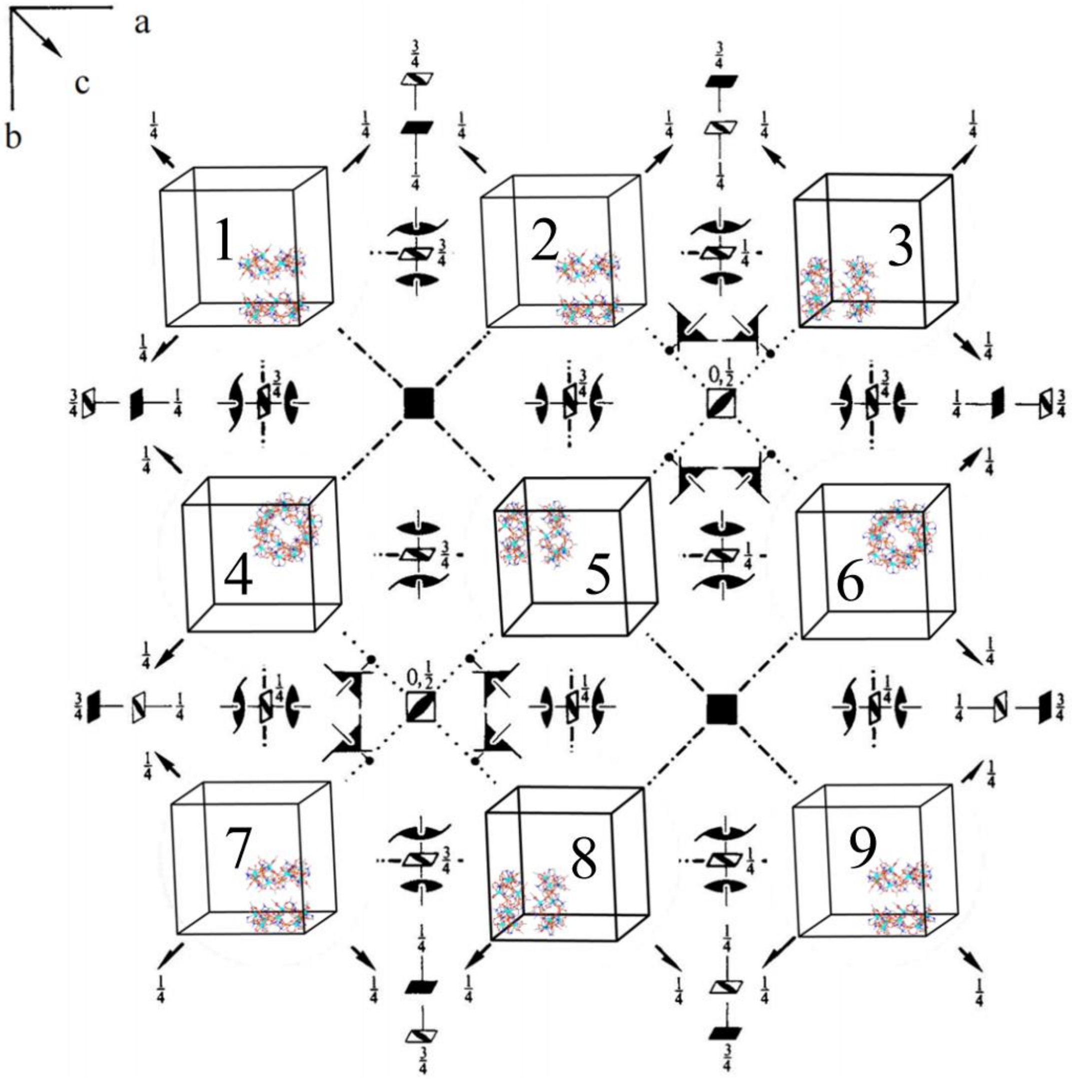

Figure S3. The International Space Group Table definition of molecular packing.

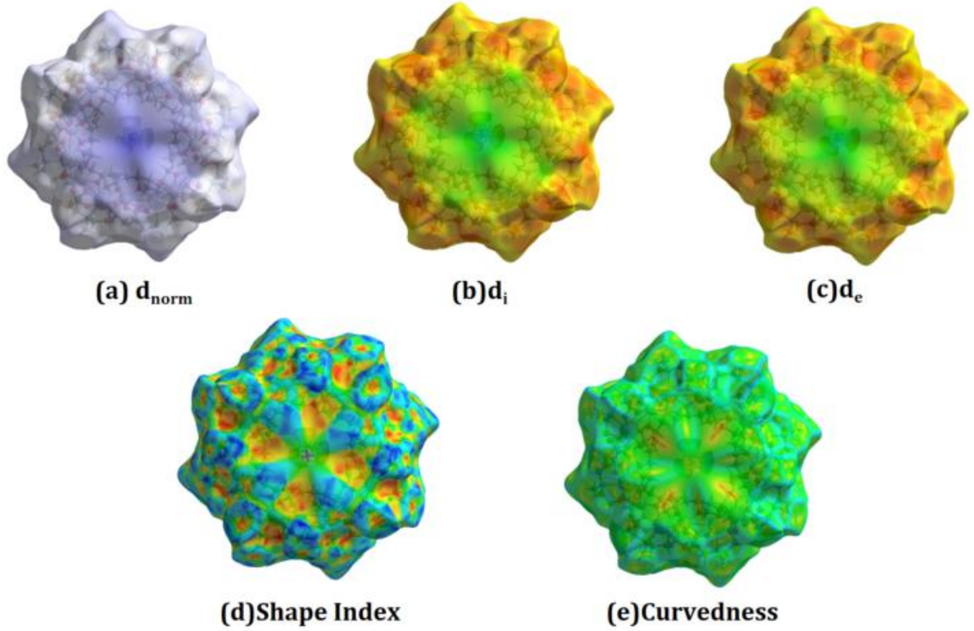

Figure S4. Hirshfeld surface mapped with (a) d_{norm}, (b) d_i, (c) d_e, (d) shape index and (e) curvedness for Fe_8Gd_8.

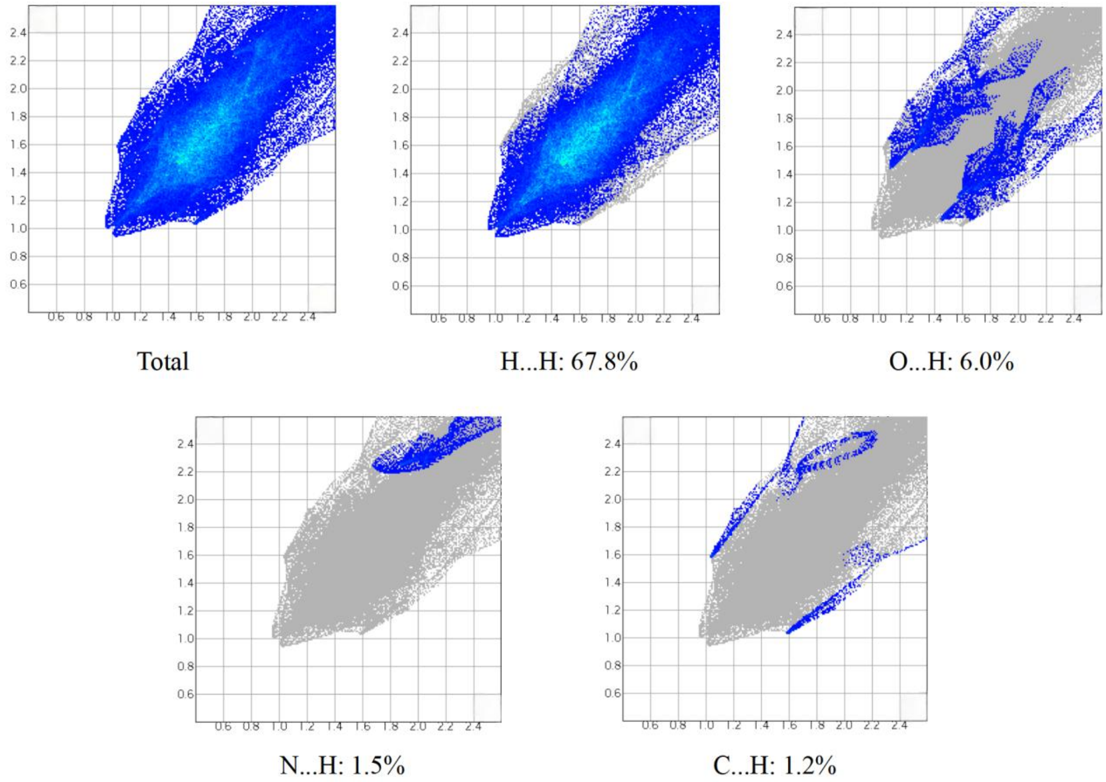

Figure S5. Fingerprint plots of Fe_8Gd_8 showing interactions.

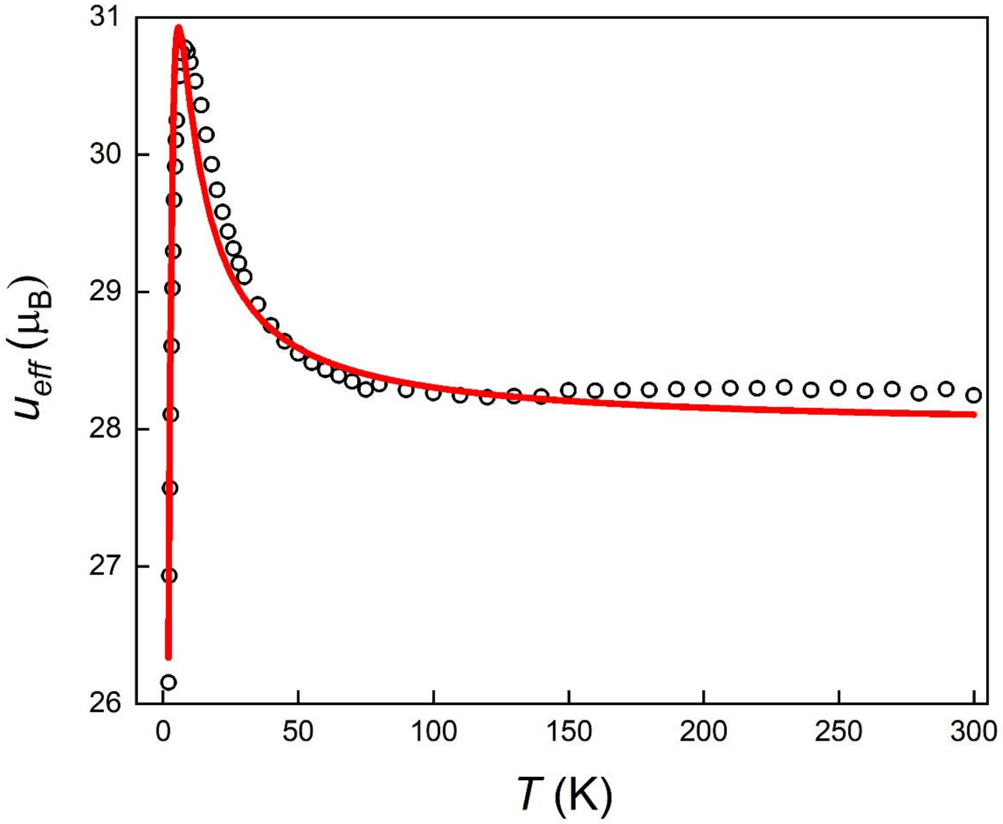

Figure S6. Temperature dependence of effective moment μ_{eff} measured under 0.1 T and red solid lines represent the best fit.

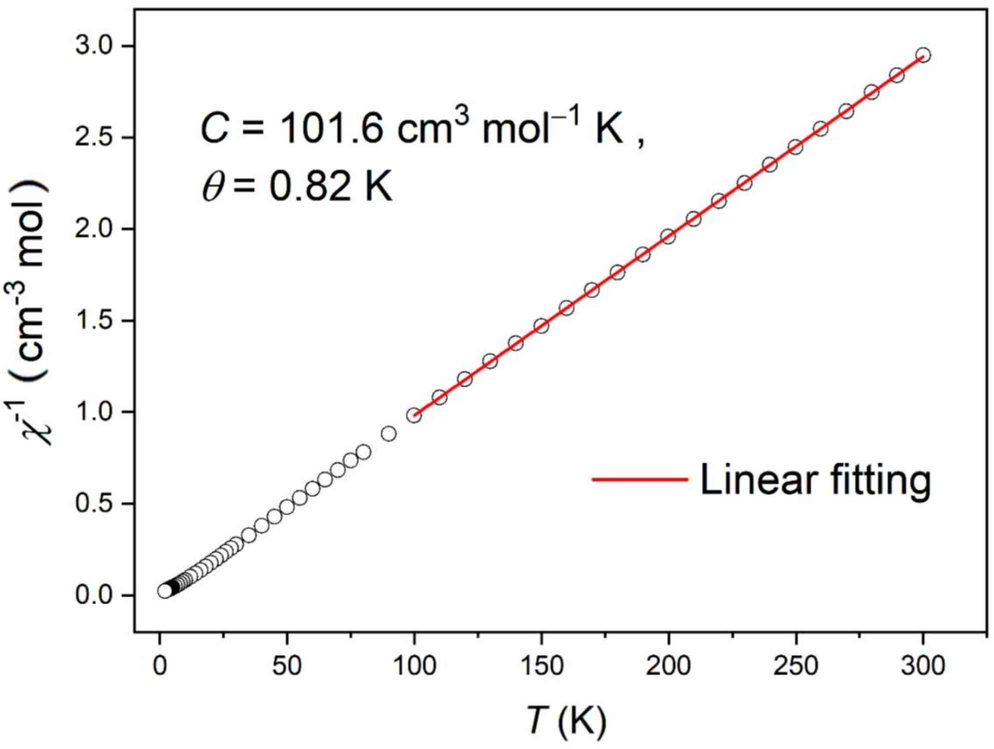

Figure S7. χ^{-1} *vs.* *T* plots for Fe_8Gd_8.

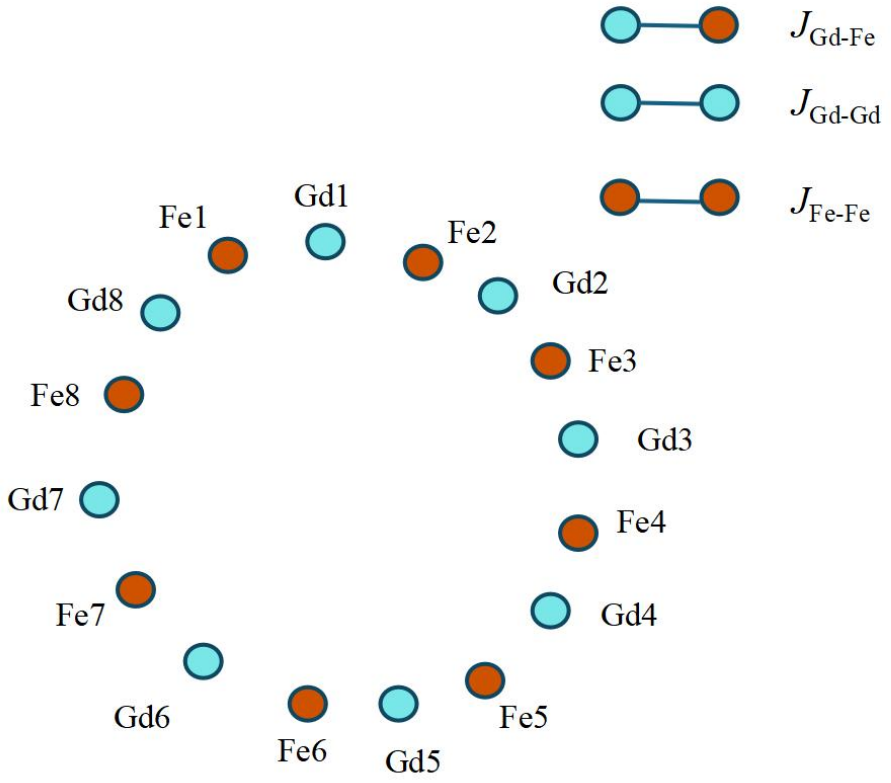

Figure S8. The metallic arrangement of Fe_8Gd_8: The specific manifestation of three magnetic exchange constants *J*.

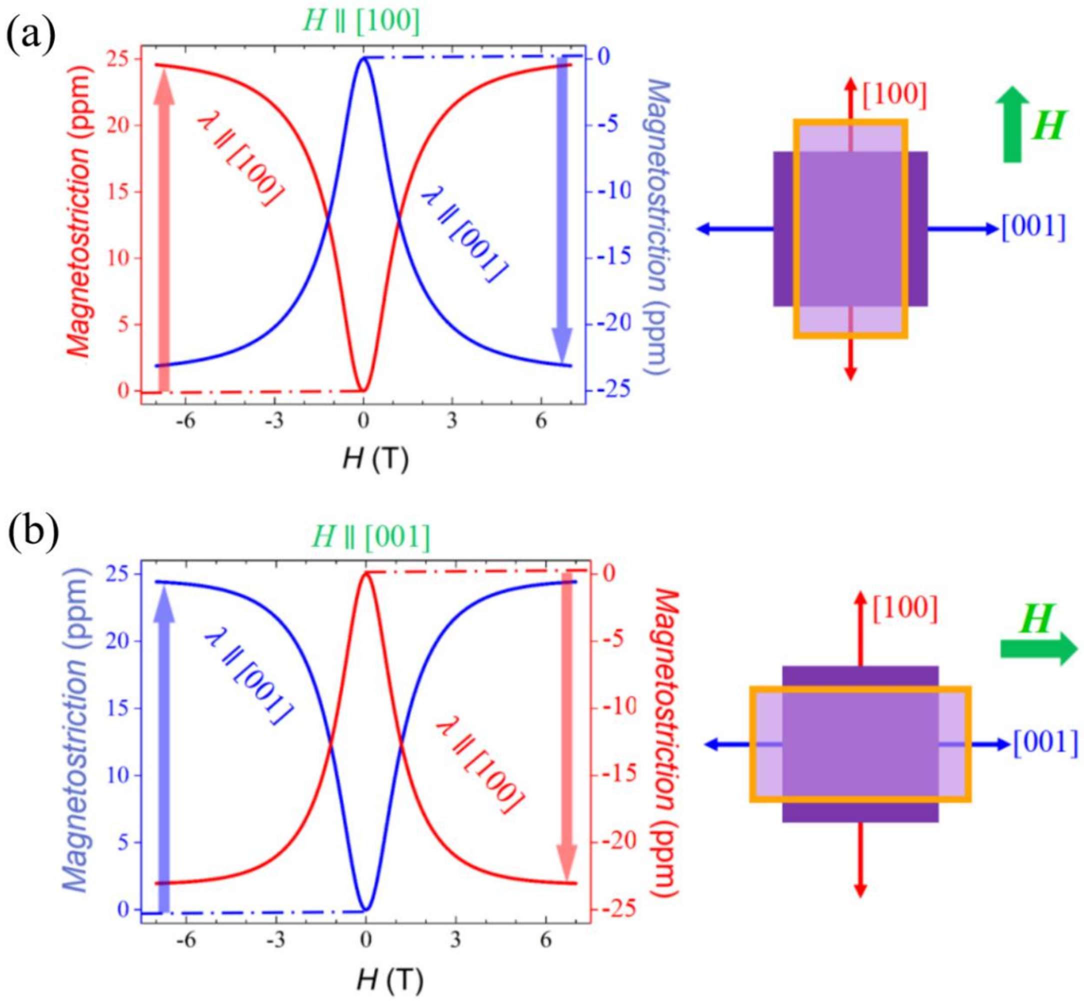

Figure. S9. Magnetostriction curve at 2K. (**a**) Magnetostriction response with the magnetic field applied parallel to the [100] direction. (**b**) Magnetostriction response with the magnetic field applied parallel to the [001] direction.

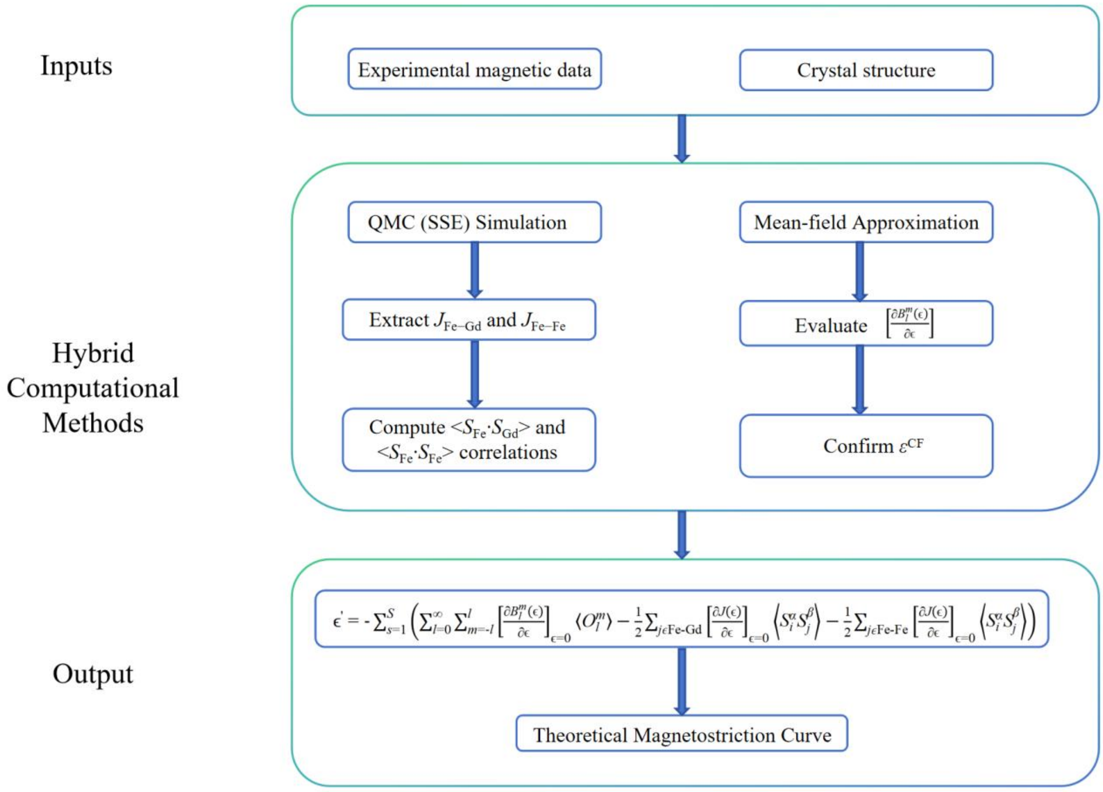

Figure S10. Computational workflow for the hybrid QMC-MFA modelling of magnetostriction.

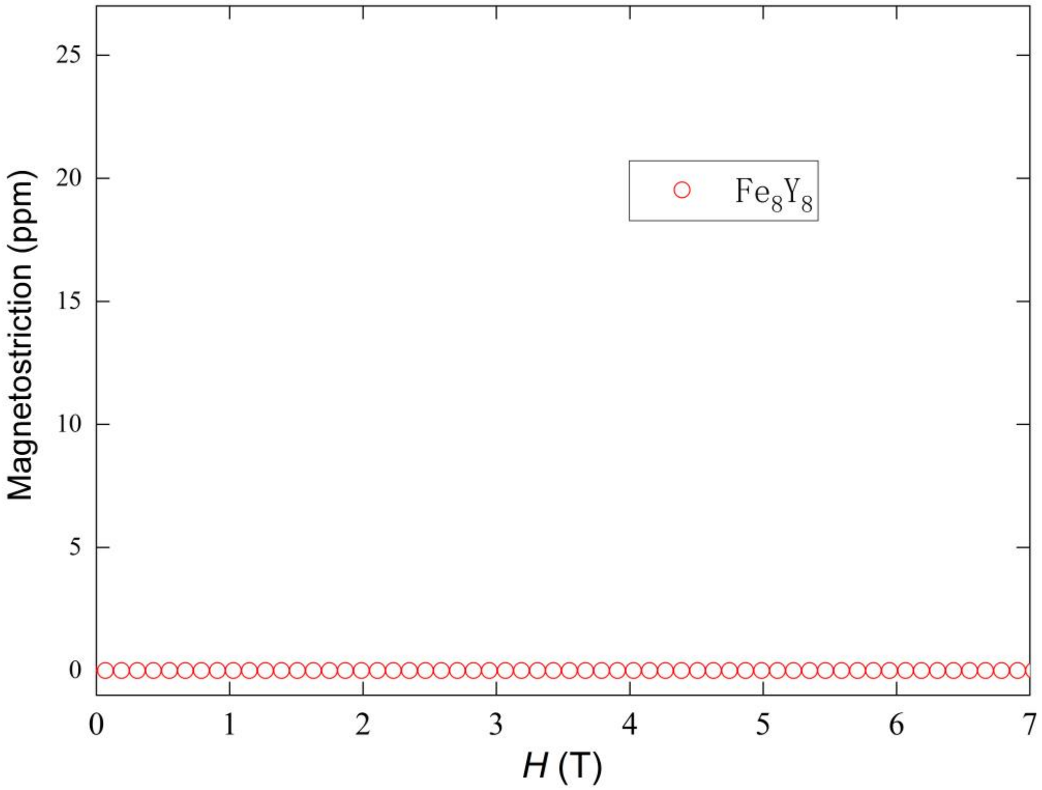

Figure S11. Magnetostriction in Fe_8Y_8 Single Crystal at 2K.

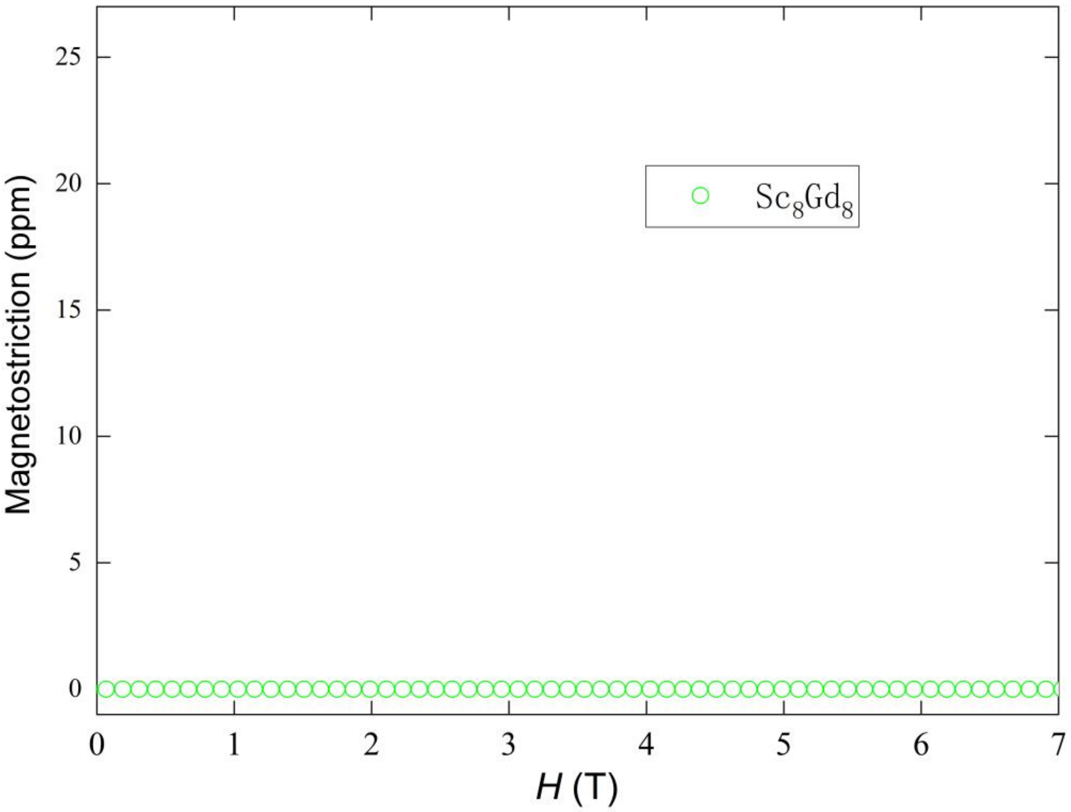

Figure S12. Magnetostriction in Sc_8Gd_8 Single Crystal at 2 K.

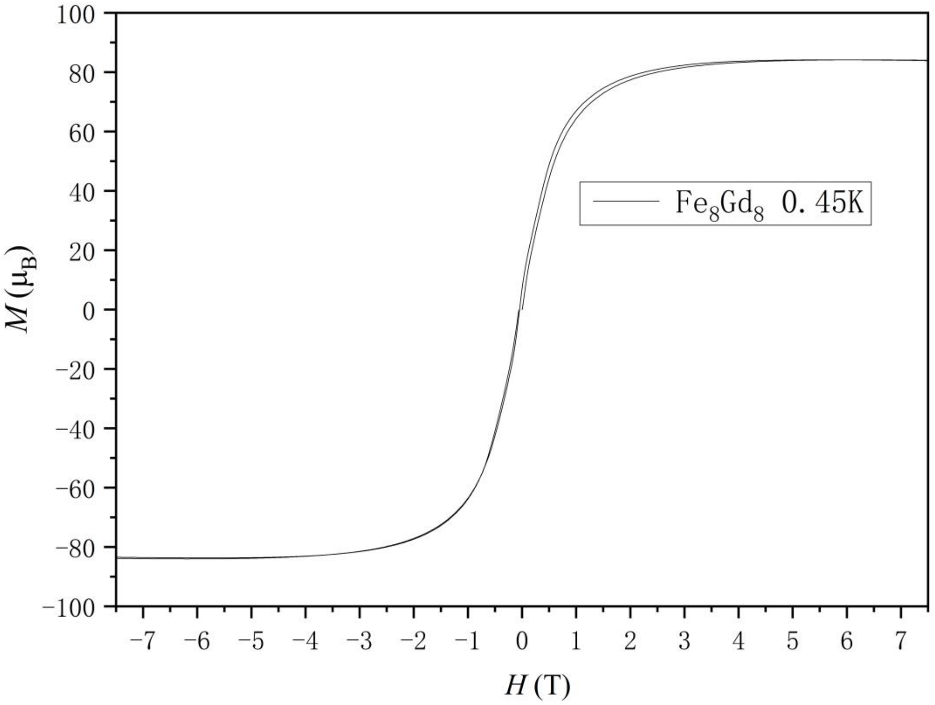

Figure S13. Pulsed-Field dependence of magnetization plots at 0.45 K.

Table S1. The MS strain of typical traditional alloys.

	Sample	MS strain / ppm		Sample	MS strain / ppm
Transition metals	Fe	21	Lanthanide metal	Dy	1400@78 K
	Ni	-46	Alloy with lanthanide doping	$Tb_{70}Fe_{30}$	1590
Metallic oxide	<111>Fe_3O_4	-78		$SmFe_2$	-1590
Alloy	$Co_{60}Fe_{40}$	68		$TbFe_2$	1753
	$Fe_{49}Co_{49}V_2$	70		<110>$Tb_{0.3}Dy_{0.7}Fe_{1.95}$	1500-2000
	<100>$Fe_{83}Ga_{17}$	400		<100>$Dy_3Fe_5O_{12}$	-1400@4.2 K
	<100>$Ti_{0.56}Fe_{2.44}O_4$	179		<110>$Tb_3Fe_5O_{12}$	2420@4.2 K
	$Co_{0.8}Fe_{2.2}O_4$	-590		<100>$Ho_3Fe_5O_{12}$	-1400@4.2 K

Table S2. Crystal data and structure refinement for Fe_8Gd_8.

Compound	Fe_8Gd_8
Formula	$C_{114}H_{247}N_{17}Fe_8Gd_8O_{74}$
Fw (g mol^{-1})	4745.01
T (K)	100(2)
Crystal system	Cubic
Space group	$Pn\bar{3}n$
a (Å)	39.872(4)
b (Å)	39.872(4)
c (Å)	39.872(4)
α (deg.)	90
β (deg.)	90
γ (deg.)	90
V (Å^3)	63389(10)
Z	12
D (g cm^{-3})	1.435
μ (mm^{-1})	3.07
Goodness-of-fit on F^2	1.183
R_1[a]	0.0938
wR_2[b]	0.2064

[a] $R_1 = \Sigma||F_o| - |F_c||/\Sigma|F_o|$, [b] $wR_2 = [\Sigma w(F_o^2 - F_c^2)^2/\Sigma w(F_o^2)^2]^{1/2}$

Table S3. Selected bond lengths [Å] for Fe_8Gd_8.

Gd(1)-O(2)	2.343(7)	Fe(1)-O(1)	1.996(7)
Gd(1)-O(3)	2.357(7)	Fe(1)-O(2)	1.982(7)
Gd(1)-O(4)	2.276(9)	Fe(1)-O(3)	1.958(7)
Gd(1)-O(5)	2.441(8)	Fe(1)-O(14)#1	2.026(8)
Gd(1)-O(6)	2.441(8)	Fe(1)-O(16)#1	1.987(7)
Gd(1)-O(7)	2.419(7)	Fe(1)-N(1)	2.251(10)
Gd(1)-O(9)	2.327(7)	Fe(2)-O(4)	1.977(7)
Gd(1)-N(2)	2.689(8)	Fe(2)-O(8)	2.059(7)
Gd(2)-O(1)#2	2.317(7)	Fe(2)-O(9)	1.985(7)
Gd(2)#1-O(1)	2.317(7)	Fe(2)-O(10)	1.992(7)
Gd(2)-O(10)	2.346(7)	Fe(2)-O(15)	1.954(6)
Gd(2)-O(11)	2.443(7)	Fe(2)-N(3)	2.276(9)
Gd(2)-O(12)	2.477(8)		
Gd(2)-O(13)	2.401(7)		
Gd(2)-O(16)	2.335(7)		
Gd(2)-N(4)	2.684(9)		
